# Removal of the Sinusoidal Transorbital Alternating Current Stimulation Artifact From Simultaneous EEG Recordings: Effects of Simple Moving Average Parameters

**DOI:** 10.3389/fnins.2020.00735

**Published:** 2020-07-29

**Authors:** Małgorzata Żebrowska, Piotr Dzwiniel, Wioletta Joanna Waleszczyk

**Affiliations:** ^1^Laboratory of Visual Neurobiology, Nencki Institute of Experimental Biology of the Polish Academy of Sciences, Warsaw, Poland; ^2^Faculty of Physics, Warsaw University of Technology, Warsaw, Poland

**Keywords:** EEG, non-invasive electrical stimulation, transorbital alternating current stimulation, sinusoidal stimulation, stimulation artifact, simple moving average, averaging parameters, artifact template removal

## Abstract

Alternating current stimulation is a promising method for the study and treatment of various visual neurological dysfunctions as well as progressive understanding of the healthy brain. Unfortunately, due to the current stimulation artifact, problems remain in the context of analysis of the electroencephalography (EEG) signal recorded during ongoing stimulation. To address this problem, we propose the use of a simple moving average subtraction as a method for artifact elimination. This method involves the creation of a template of the stimulation artifact from EEG signal recorded during non-invasive electrical stimulation with a sinusoidal alternating current. The present report describes results of the effects of a simple moving average filtration that varies based on averaging parameters; in particular, we varied the number of sinusoidal periods per segment of the recorded signal and the number of segments used to construct an artifact template. Given the ongoing lack of a mathematical model that allows for the prediction of the “hidden” EEG signal with the alternating current stimulation artifact, we propose performing an earlier simulation that is based on the addition of artificial stimulation artifact to the known EEG signal. This solution allows for the optimization of filtering parameters with detailed knowledge about the accuracy of artifact removal. The algorithm, designed in the MATLAB environment, has been tested on data recorded from two volunteers subjected to sinusoidal transorbital alternating current stimulation. Analysis of the percentage difference between the original and filtered signal in time and frequency domain highlights the advantage of 1-period filtration.

## Introduction

Transorbital and transcorneal alternating current stimulation appear to be some of the most promising tools for studying and the rehabilitation of visual dysfunctions. As a result, there is a recent shift in the use of these methods from research laboratories to clinics (for review see Ota et al., 2018; Sabel et al., 2019). Detailed analysis of the brain activity during stimulation is crucial for elucidating the processes underlying the generation of phenomena associated with mentioned stimulation.

**FIGURE 1:**
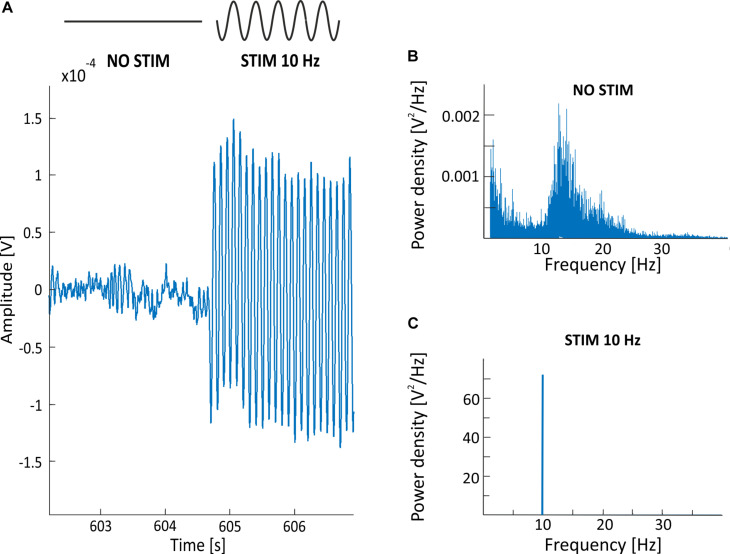
**(A)** Example of the EEG recording (electrode O2; one exemplar participant) with a visible stimulation artifact starting around 604.5 s and resulting from an applied 40 μA 10 Hz sinusoidal to ACS. The EEG signal with superimposed stimulation artifact is 10 times greater in amplitude than the EEG signal recorded prior to stimulation. **(B)** Power spectrum density (PSD) before stimulation in a condition with an excitation in alpha band (i.e., closed eyes). The large intensity of artifact in 10 Hz during stimulation **(C)** doesn’t allow for the analysis of the spectrum of endogenous brain activity in alpha band. PSD obtained from 5 min of signals, recorded at a 500 Hz sampling frequency.

There are few possible types of alternating current stimulation (ACS) wave shapes, including sinusoidal, triangle/sawtooth, and squared (Moreno-Duarte et al., 2014; Dowsett and Herrmann, 2016). In this work, we focus on the sinusoidal transorbital (to-) ACS given that the usefulness of this method (as well as other types and subtypes of ACS) in rehabilitation of visual dysfunction is unknown (Kanai et al., 2008; Brignani et al., 2013; Neuling et al., 2013; Vossen et al., 2015; Kasten et al., 2016). Our main concern is that analysis of the EEG signal recorded during stimulation is significantly impeded due to the presence of a stimulation artifact that completely obscures endogenous brain activity. A representation of this problem is provided in Figure 1, showing signal recorded during 40 μA 10 Hz sinusoidal toACS. The amplitude of the signal with toACS stimulation is much greater than the amplitude of the signal recorded prior to stimulation in both time (Figure 1A) and frequency domains (Figures 1B,C). This problem is complicated by the fact that the frequency of stimulation usually falls within the frequency of interest, which is crucial for the study of a given phenomenon. With regard to these problems, the conclusions about the impact of a particular stimulation protocol are usually drawn based on a comparison of the EEG signal recorded before and after stimulation. Knowledge about the EEG signal “hidden” under the artifact could provide additional information on the impact of stimulation on neural activity and thus enable scientists to extend and refine findings on the impact of stimulation. Therefore, a method of filtering the EEG signal is needed, allowing for the removal of artifact with the greatest possible accuracy while ensuring the smallest possible loss of information about endogenous brain activity.

The search for the most appropriate method of removing ACS artifacts initially begin in studies concerning functional magnetic resonance imaging (fMRI). The ACS artifact observed in the EEG signal is similar to the artifact recorded during fMRI with simultaneous stimulation. The previously proposed methods of filtration were based on a combination of average (i.e., template) artifact subtraction and other techniques, such as adaptive noise cancelation (Allen et al., 2000), principal component analysis (PCA; Niazy et al., 2005), or independent component analysis (ICA) with different filtering (Bénar et al., 2003). Some of these methods have already been used to remove the ACS artifact. Template subtraction and PCA have been adapted to remove 10 Hz sinusoidal tACS artifact (Helfrich et al., 2014) using a 2-step algorithm. This algorithm involves the calculation of an artifact template from artifact segments. Obtained artifact template is then subtracted from a segmented signal and the remaining artifact is subsequently removed with the use of a PCA. The combination of average template subtraction and PCA was also used by Kohli and Casson (2015). In this study, both techniques were used independently and their filtering results were compared.

Previous publications that discuss the removal of the ACS artifact have provided only brief explanations of the adopted filtration parameters. We have found insufficient explanation of the impact of mentioned filtration settings on the overall filtering procedure outcomes. These publications will be discussed briefly in the following. First, in Helfrich et al. (2014), the filtering approach was based on the use of 30 period segments, and then creating a template from 10 centered segments (i.e., 300 sinusoidal periods averaged to create 1-period artifact template). Other research (e.g., Kohli and Casson, 2015) suggests that individual segments should consist of the smallest possible number of oscillation periods and that the length of the segment (i.e., number of samples) should be an integer. The number of averaged segments was equal to 5% of all segments extracted from the signal. Given (1) the lack of a clear and well-established consensus regarding the choice of the optimal averaging parameters for satisfactory stimulation artifact removal, as well as (2) the need for analysis of the relation between those parameters and the removal method performance (Kohli and Casson, 2019), we decided to consider this issue.

The present study aimed to provide a comprehensive analysis of the effectiveness of the method for sinusoidal ACS artifact removal based on the subtraction of the artifact template created with the use of simple moving average (SMA). Our aim was to propose a guide for researchers using SMA method for cleaning EEG signals that have been contaminated with sinusoidal ACS artifacts. Although the algorithm used to create an average artifact template was applied in some of the aforementioned studies, our analysis of the literature revealed insufficient information about the values of the parameters used in the averaging procedure. Furthermore, this method is often used as the only filtration step or is used as the initial step before further filtration stages (e.g., PCA, ICA). Thus, the considerations discussed here are important for the accurate separation of the EEG signal of interest from contamination due to stimulation artifact. Therefore, this work focuses on illustrating the effects of various SMA parameters and their respective values on the performance of stimulation artifact removal from the EEG signal recorded during sinusoidal toACS. We included a quantitative analysis of the accuracy of the stimulation artifact removal and an assessment of the pros and cons of using SMA filtering in the EEG signal analysis.

## Materials and Methods

### Ethical Approval

The study adhered to the Declaration of Helsinki and was approved by the Ethical Committee of the University of Warsaw. Participants provided written informed consent concerning their participation in the study.

### Subjects

One healthy female (age 23 years) and one healthy male (age 30 years) participated in the study. The participants were co-authors of this article and were required to meet the following criteria: (1) age between 20 and 40 years old; (2) lack of myopia or other uncorrected visual acuity deficits; (3) lack of diagnosed mental disorders; (4) lack of diagnosed neurological diseases or disorders; (5) lack of history of epileptic seizures; (6) lack of history of head injury resulting in loss of consciousness and/or hospitalization with comorbid brain damage; (7) lack of intake of psychoactive substances including medical drugs; (8) lack of diagnosed addiction to any psychoactive substance; (9) lack of implanted electronic devices; (10) not pregnant.

### Study Preparation

Prior to the experiment, each participant was appropriately prepared for the study. First, we applied the EEG cap on the participant’s head. We ensured good contact between the EEG recording electrodes and the participant’s skin via SuperVisc conductive gel (EASYCAP, Germany). Next, the participant’s skin was cleaned and hydrated below and above both eyes with the use of 70% ethanol and Nuprep skin preparation gel (Weaver and Company, United States), respectively. Finally, self-adhesive current stimulation electrodes were placed in the prepared areas around the participant’s eyes. The accepted impedance threshold between skin and electrodes was set to 10 kΩ.

### Hardware Configuration and Experimental Design

#### Visual Stimulation and EEG Recording

**FIGURE 3:**
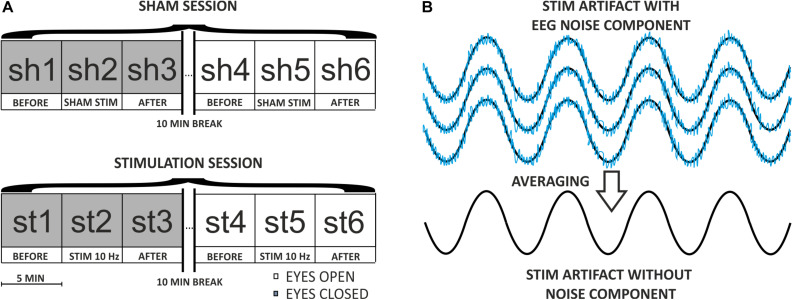
**(A)** Diagram of the experimental procedure on the first participant. The procedure consisted of two continuous 15-min blocks (one with eyes closed, one with eyes open) repeated over the course of two subsequent days (SHAM and STIMULATION). A 10-min break separated each block, and EEG data were not recorded during the breaks. During every 15-min session, the stimulation lasted 5 min, and had a frequency of 10 Hz and an amplitude of 20 μA. **(B)** Diagram of the coherent averaging idea. In this example, four noisy sinusoidal segments compatible with phase are averaged resulting in a “pure” sinusoidal signal. The noise component with a low amplitude represents endogenous EEG activity embedded in a high-amplitude sinusoidal wave representing an ACS artifact. The noise after averaging is close to 0, which indicates that coherent averaging sets information about endogenous EEG activity to zero and thus allows for the acquisition of the ACS artifact template (i.e., sinusoidal component).

The participant was situated in front of a laptop screen at a distance of 80 cm and was instructed to fixate on a white circular point (diameter: 0.3°, luminance: 207.5 cd/m^2^) displayed on a homogenous black background (luminance: 0.3 cd/m^2^). The experiment with the male participant consisted of two continuous 15-min blocks repeated over two subsequent days. On day one, EEG data were recorded while the participant’s eyes were closed for the first block, and eyes open for the second block. On day two, toACS was applied during the middle 5 min of each block while performing simultaneous EEG recording. A 10-min break separated each block and EEG data was not recorded during the breaks (Figure 3A).

EEG data was recorded from the male subject using an actiCHamp EEG amplifier, an actiCAP EEG cap equipped with 32 active recording electrodes and one additional ground electrode, and recording software (Brain Products, Germany). The ground electrode was placed at the AFz electrode location and the software reference electrode was set at Cz. Thus, the raw EEG data consisted of 31 channels, given that the reference electrode was not included. Sampling frequency was set to 10 kHz. Low- and high-pass hardware filters were 2470 Hz and DC, respectively.

Detailed information about EEG data collection for the second subject (female) is presented in Supplementary Materials.

#### Electrical Stimulation

**FIGURE 2:**
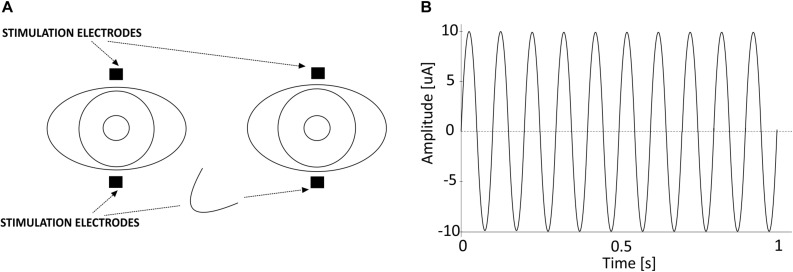
**(A)** Location of the electrical stimulation electrodes used in the study. **(B)** One second of the 10 Hz 20 μA sinusoidal alternating current stimulation that was applied transorbitally.

The generation and application of toACS was performed using DC-Stimulator MC (neuroConn, Germany). The stimulator has four stimulation output channels. The first two channels were used to apply toACS to the participant. The third channel carried the same information as the first two channels, but instead of using the channel for stimulation, it was sent via opto-isolator to the EEG amplifier’s AUX input and later used for EEG signal segmentation. Stimulation was applied via four 15 × 20 mm rectangular self-adhesive EMG electrodes (Spes Medica, Italy) connected with external cables to the stimulator and located directly below and above the participants’ eyes, i.e., transorbitally (Figure 2A). Impedance between the stimulation electrodes and participants’ skin never exceeded 100 kΩ. If the impedance exceeded 100 kΩ, the stimulator would automatically stop the procedure due to safety issues. Current stimulation was in a form of sinusoidal wave of 10 Hz frequency and 20 μA amplitude from peak-to-peak (Figure 2B). Maximum calculated current density of the applied current stimulation below each of the stimulating electrodes during stimulation peak was 0.066 μA/mm^2^. Stimulation signal generated by the stimulator was prepared in Python programming language as ^∗^.mat file, and converted with a dedicated neuroConn’s MATLAB toolbox into a ^∗^.bfs file, a file format used by the stimulator for stimulation. The sampling frequency of the stimulation signal was 16 kHz.

### Data Pre-processing

Pre-processing of the recorded EEG data was performed with the use of custom-written scripts in Python programming language and MNE-Python package (Gramfort et al., 2013; Jas et al., 2018). First, the raw EEG data were filtered with a Butterworth 4th order biquadratic (i.e., second-order sections) IIR band-pass filter for the 0.1–100 Hz frequency range. Then, data were filtered with a zero-phase FIR notch filter of length 6.6 s for grid artifact frequency and associated harmonics removal, i.e., 50, 100, 150, 200, and 250 Hz. Of note, the filter type selection was motivated by the need to minimize edge artifacts around the current stimulation EEG artifacts.

### Designed Algorithm for the Removal of the Sinusoidal ACS Artifact From EEG Signal

The algorithm that removes the sinusoidal ACS artifact from the simultaneous EEG data recording was implemented in the MATLAB environment. This algorithm is based on the coherent averaging technique, which is useful in digital signal processing to filter out noisy time series from repetitively applied stimuli (Rompelman and Ros, 1986). This method assumes that noise components are additive. Typically, during such filtration, the signal of interest is a periodic wave (e.g., a sinusoidal function) that is embedded in the noise. Averaging the corresponding noisy signal segments that are compatible in the phase causes the noise to be cleared, thus increasing the signal-to-noise ratio. In the case of EEG signal with embedded sinusoidal ACS artifact, the situation appears to be exactly the opposite (see Figure 3B).

The unwanted ACS component is a sinusoidal waveform of a specific frequency. Averaging the appropriate number of sinusoidal periods results in a filtered waveform. This waveform serves as a template for artifact that can later be subtracted from the recorded signal (Figures 3A,B). Due to the specificity of the EEG signal recorded during stimulation, the part of the signal that should be averaged to obtain the most comprehensive artifact template is not readily apparent. In fact, the artifact embedded in the signal may change over time due to possible impedance changes at the skin-electrode interface caused by sweating, peeling off the electrodes, or drying of the conductive gel. One possible solution to the progressive changes related to these potential fluctuations of the electric potential on the skin-electrode interface is the application of a coherent moving average (also called SMA) with a defined window that limits the range of the averaged signal. The algorithm, described in detail in the next paragraph, can be used to filter one-dimensional continuous time series; for example, recordings from a single EEG electrode.

#### Steps of the Algorithm

##### Division of the signal into segments

Knowing the sampling frequency of the signal *F*_*s*_ and the frequency of the stimulation *freqStim*, the number of samples corresponding to the length of the segment containing one full sine wave stimulation period can be calculated as follows:

(1) $$s\,e\,g\,m\,e\,n\,t\,L\,e\,n\,g\,t\,h=\frac{F_{s}}{f\,r\,e\,q\,S\,t\,i\,m}$$

wherein *segmentLength* is the number of samples that corresponds to the length of one stimulation period.

**FIGURE 4:**
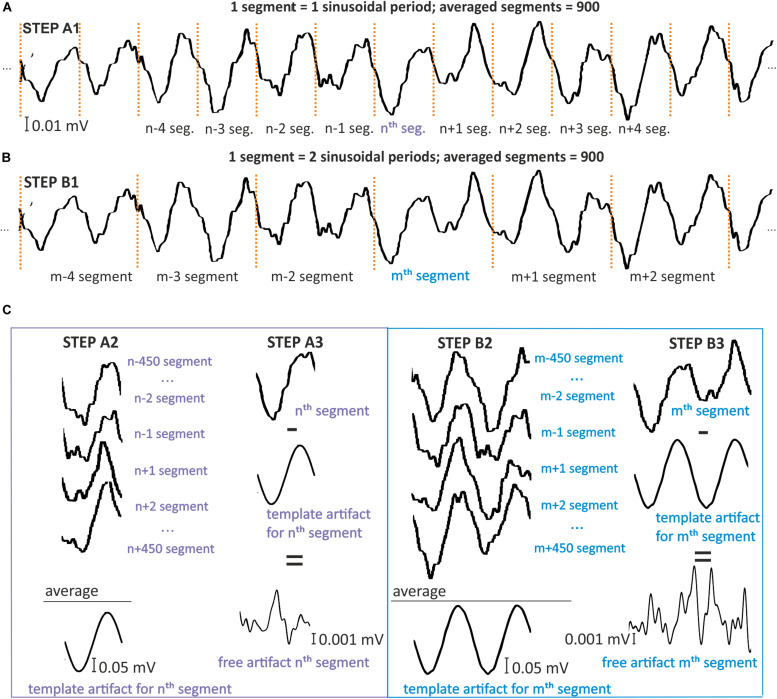
Model for simple moving average filtration for 1- **(A)** and 2-period **(B)** segments. Simulation was prepared on signal STEP A1, B1, by dividing the signal for one or two period segments [s(n) and s(m)]. STEP A2, B2: To create an artifact template for the nth or mth segment, 900 other segments were selected that centered around the considered segments. The samples in the same phase were then averaged by columns. STEP A3,B3: The resulting artifact templates were then subtracted from the nth and mth segments that included artifact. This subtraction results in nth and mth segments that are free of artifact.

Starting with the first sample that includes stimulation, the signal is divided into single-period segments (Figure 4A) or into segments of a total multiplicity of the period (Figure 4B). Each segment has the same length, corresponding to the same number of samples. As a result, the signal with the stimulation artifact, arising from one electrode (*E*), consists of segments *s*(*n*) of the same length, according to the following rule:

(2) E=[s⁢(1),s⁢(2),s⁢(3),…,s⁢(N)]

wherein *s*(*n*) represents successive segments, i.e., the sets of samples containing the total number of stimulus oscillations and *n* ∈ < 1:*N* >, *N* is total number of segments.

##### Calculation of the artifact template

For each segment, *s*(*n*), the artifact template *temp* is calculated on the basis of *A*.*A*refers to the number of averaged segments centered around the segment *s*(*n*). In this process, the segment that is used to count the template is not used in averaging to avoid any possible later subtraction of information about the pure EEG. For this reason, the artifact template *temp*(*n*) for the segment*s*(*n*) is determined according to the following relationship:

(3) $$t\,e\,m\,p\,\left(n\right)=\frac{1}{A}\,\left[\sum_{n+1-\frac{A}{2}}^{n+1+\frac{A}{2}}s\,\left(n\right)\right]$$

wherein *temp*(*n*) represents an artifact template for segment *s*(*n*), *A* represents an even number of averaged segments, *s*(*n*) represents the segment, *n* ∈ < 1:*N* >, and *N* refers to the total number of segments.

##### Subtraction of templates from segments

The last stage consists of subtracting the prepared templates from the corresponding segments. This operation results in a new segment, *newS*(*n*), that is free of artifact and can be calculated as follows:

(4) n⁢e⁢w⁢S⁢(n)=s⁢(n)-t⁢e⁢m⁢p⁢(n)

wherein *newS*(*n*) refers to a new segment without stimulation artifact, *s*(*n*) represents the artifact segment, and *temp*(*n*)represents the artifact template for segment *s*(*n*).

**FIGURE 5:**
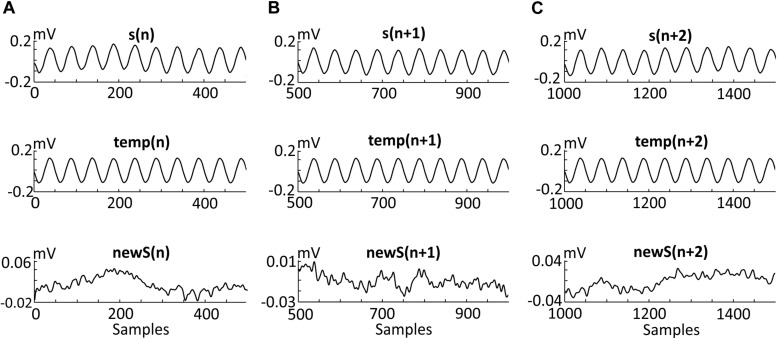
Effects of the three steps of the algorithm. Three adjacent segments [s(n) **(A)**, s(n + 1) **(B)**, and s(n + 2) **(C)**] recorded during the stimulation of the second participant (details provided in the “Methods and Materials” section). Each segment contains 10 oscillations of a 10 Hz alternating current. Thus, each segment is a 1-s recording and, due to the 500 Hz sample frequency, every s(n) contains 500 samples. Of note, one period contains 50 samples according to the simple calculation (500 Hz)/(10 Hz) = 50. Each template [i.e., temp (n), temp(n + 1), and temp(n + 2)] was created by averaging 20 segments centered around segments s(n), s(n + 1), and s(n + 2). Three new segments [i.e., newS(n), newS(n + 1), and newS(n + 2)] were obtained after subtracting the artifact templates as follow: s(n)-temp(n) = newS(n).

Figure 5 presents three consecutive example segments. Each segment [i.e., *s*(*n*), *s*(*n* + 1), and *s*(*n* + 2)] (Figures 5A–C) was obtained from a signal with 10 Hz stimulation. Each segment was constructed from 10 sinusoidal periods. Thus, each segment is a 1-s recording and, due to the 500 Hz sample frequency, every *s*(*n*) contains 500 samples. One period contains 50 samples according to the simple calculation $\frac{500\,H\,z}{10\,H\,z}=50.$ Three consecutive artifact templates [*temp*(*n*), *temp*(*n* + 1), and *temp*(*n* + 2)] were constructed from the average of 20 segments centered around the considered segment. The final effect of the filtering algorithm using the moving average method presents new filtered segments without artifact templates [*newS*(*n*), *newS*(*n* + 1), and *newS*(*n* + 2)], and with an amplitude that does not exceed 0.1 mV. The amplitude before and after the filtering has been changed more than 10 times.

#### Method for Selecting the Optimal Parameters of Filtration

Here, we analyzed the accuracy of artifact removal using the designed algorithm to verify, in detail, the effects of the two filtration parameters: (1) the number of periods per segment (i.e., in each artifact template) and (2) the number of averaged segments used to create each artifact template.

**FIGURE 6:**
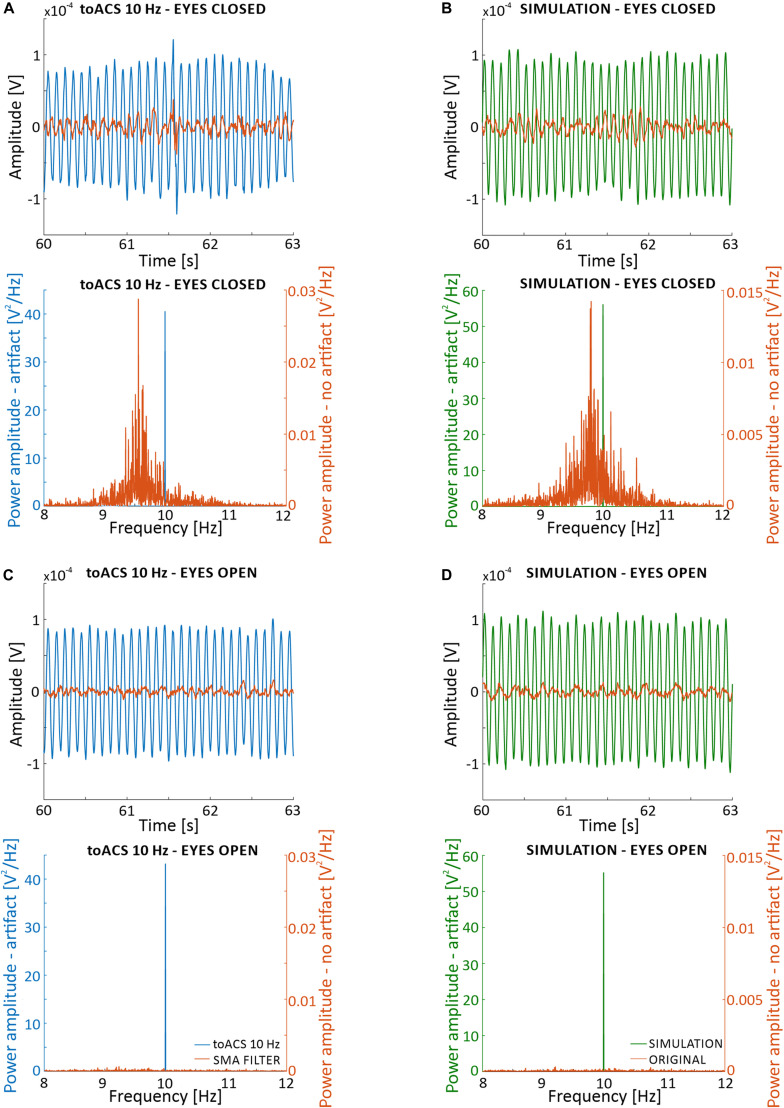
Effects of simple moving average (SMA) filtration on 5-min of EEG signal recorded from the O2 electrode from the first participant with real stimulation artifact (i.e., blocks *st2* and *st5*) **(A,C)**, and with a superimposed artificial sinusoidal function (i.e., blocks *sh2* and *sh5*) **(B,D)**. Parameters of the filtration are: 1-period segments and 900-segment artifact templates. **(A)** Time and frequency analysis for the eyes closed condition with real stimulation artifact. **(B)** Time and frequency analysis for the eyes closed condition with superimposed artificial sinusoid. **(C)** Time and frequency analysis for the eyes open condition with real stimulation artifact. **(D)** Time and frequency analysis for the eyes open condition with superimposed artificial sinusoid. The superimposed artificial sinusoid used in **(B,D)** was of 10 Hz frequency and 0.0002 V amplitude.

Verifying the effectiveness of artifact removal without simultaneously removing information about neural activity from the EEG signal is difficult due to the lack of a model that predicts the “real” EEG signal “hidden” under the artifact and lays in the middle of the inverse problem. To facilitate understanding the problem of removing stimulation artifact from EEG recordings, we present results of SMA filtering on signal recorded during eyes closed (Figure 6A) and eyes open (Figure 6C) conditions. These results are shown in time and frequency domains only, for one of several possible filtering settings (i.e., 900 1-period segments to create 1-period artifact template). Following filtration, a decrease in signal amplitude is observed in both time and frequency domains. Unfortunately, because we are unable to compare these results to a known stimulus signal, we have no information on the accuracy of removing the artifact and possible loss of information about neuronal activity. Therefore, it is important to identify the filtration conditions that would be most effective, and how to achieve a reliable assessment of the filtration algorithm.

Taking these factors into consideration, we adopted the following principle. First, a sinusoidal waveform interpolated and downsampled to simulate an undefined sinusoidal waveform in the actual EEG recording, *EEG*_*S*_, was superimposed on pure *EEG* signal with a known power spectrum. Then, after applying the algorithm with different filtering parameters, the obtained signal (*EEG*_*removedArtifact*_) was compared with the original pure *EEG* signal. The described algorithm is as follows:

1. *EEG* + *SINUS* interpolated by the order of 10, then subjected to the downsampled by the order of 10 – (*EEG*_*S*_).
2. *EEG*_*S*_ subjected to the sine artifact removal algorithm – (*EEG*_*removedArtifact*_).
3. Compare *EEG* with *EEG*_*removedArtifact*_.
  wherein *EEG* represents the EEG signal from the selected electrode, *SINUS* refers to the artificial stimulation in a form of sinusoidal function with a known frequency (*freqStim*), and amplitude (*Amp*).

Performing the simulation (see Figures 6B,D) using this approach allows for the determination of the optimal filtration parameters. These optimal parameters could then be used on the signal recorded during a real stimulation session. Upon application to real data, it is important that the EEG signal used in the simulation has the same sampling frequency and duration. Also, the artifact should be characterized by the frequency given in the actual stimulation. The number of averaged oscillations needed to create an artifact template is increased gradually until the signal is the closest to the original recorded signal. The resulting fixed length of the averaging window could then be used to remove the artifact in the stimulation signal.

To perform filtration of blocks *st2* (i.e., signal with eyes closed during stimulation, Figure 3A), a simulation needs to be prepared using a signal from block *sh2* (i.e., eyes closed during sham stimulation). The choice of signals is dictated by the need to preserve a similar condition of the subject from the real stimulation stage and the signal without the stimulation that is used to perform the simulation. For this reason, an analogous approach should be used to filter the block *st5* (i.e., signal with eyes open during stimulation). The reference signal for this case is block *sh5* (i.e., signal with eyes open during sham stimulation). In our opinion, it is important to keep the characteristics of the reference signals as close as possible to the signal from which the artifact should be removed. The simulation of artifact removal from block *sh2* contained the following components: (1) *EEG* (signal from the O2 electrode, block lasting 5 min, eyes closed during sham stimulation) and (2) *SINUS* (artificial stimulation, a sinusoidal function with *freqStim* = 10 Hz, and *Amp* = 0.0002 V (peak-to-peak of the sine wave).

To determine the optimal averaging parameters, we evaluated the percentage difference in the power spectrum (i.e., spectrum percentage difference, SPD) in the alpha band, and the difference in stimulation frequency between the original and filtered signal. The SPD was calculated as follow (5):

(5) $$\begin{array}{l} differenc\;ebetwee\;nspectr\;a(SPD)\\ =\frac{\sum_{f=f}^{F}|P_{pureEEG}-P_{removedArtifact}|}{\sum_{f=f}^{F}|P_{pureEEG}|}*100 \end{array}$$

wherein *P*_*removedArtifact*_ is the power of the signal after filtration; *P*_*pureEEG*_ is the power of the original signal; and f and F represent the minimum and maximum frequencies of the band, respectively.

The value of the signal’s power in the given frequency band from *f* to *F* was determined using the following Formula (6):

(6) $$P_{p\,u\,r\,e\,E\,E\,G/r\,e\,m\,o\,v\,e\,d\,A\,r\,t\,i\,f\,a\,c\,t}={\left|F\,F\,T\,\left(d\,f\right)\right|}^{2}$$

where in *FFT*(*df*) is the value of the signal amplitude calculated using the Fast Fourier Transform algorithm for frequencies from *f* to *F* with the frequency resolution *df*.

SPD in alpha band was calculated in the 8–12 Hz range, and in stimulation of 10 Hz frequency in the range of 9.5–10.5 Hz.

An analogous approach was used to analyze the differences in signals in the time domain by determining the differences in signal variances. Here, the variance of the time series is understood as a measure of the fluctuation of the signal amplitude values around the mean value. The variance, σ^2^, of the time series *x*(*n*) defines the following relationship (7):

(7) $$\sigma^{2}=\frac{1}{N}\,\sum_{n=1}^{N}{\left(x\,\left(n\right)-x_{a\,v\,e\,r\,a\,g\,e\,d}\right)}^{2}$$

wherein *N* is the number of samples in *x*(*n*), and *x*_*averaged*_ is the average value of amplitude in *x*(*n*).

The difference between signal variances was calculated according to the following relationship (8):

(8) $$\begin{array}{l} difference\;between\;variances\\ =\frac{{\text{σ}}_{pureEEG}^{2}-{\text{σ}}_{removedArtifact}^{2}}{{\text{σ}}_{pureEEG}^{2}}*100 \end{array}$$

wherein $\sigma_{p\,u\,r\,e\,E\,E\,G}^{2}$ is the variance of the original signal, and $\sigma_{r\,e\,m\,o\,v\,e\,d\,A\,r\,t\,i\,f\,a\,c\,t}^{2}$ is the variance of the signal after filtration.

## Results

### Differences in the Frequency Domain

**FIGURE 7:**
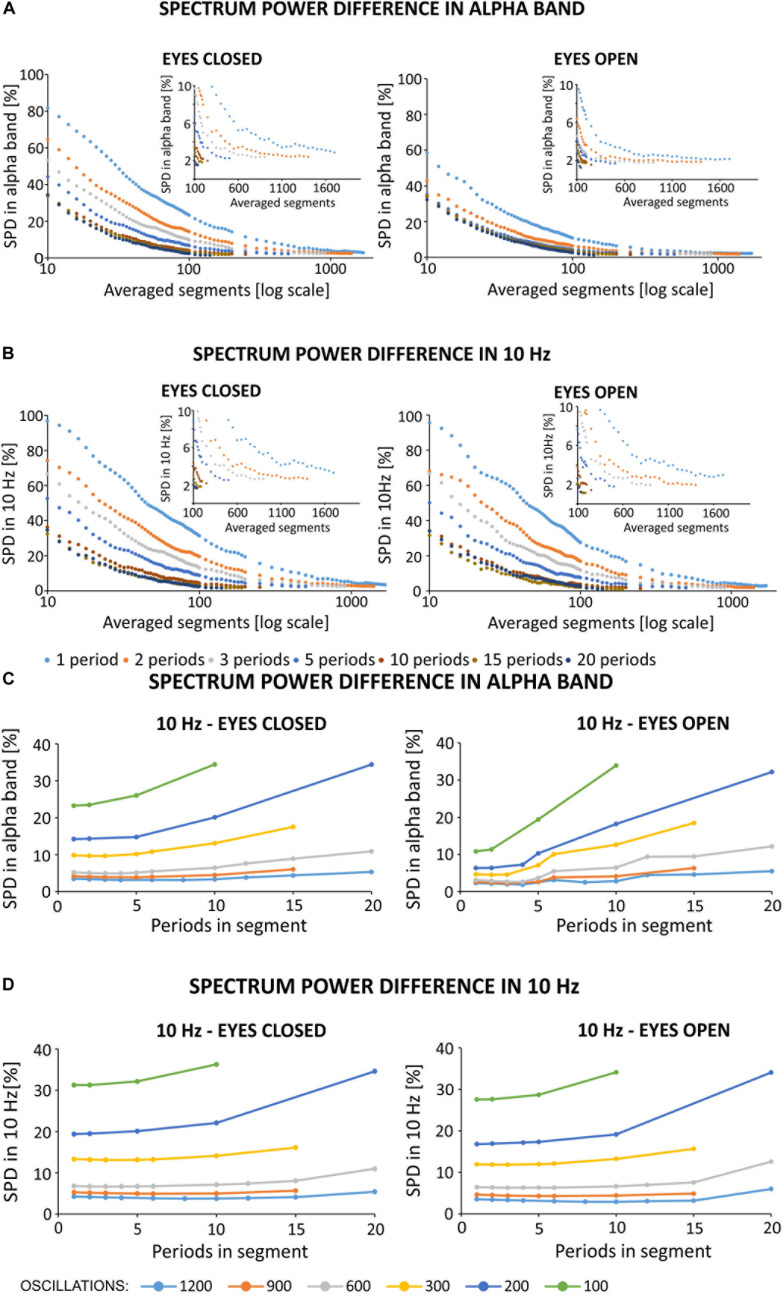
**(A,B)** Spectrum percentage difference (SPD) between the original signal and the signal obtained after removing the simulated 10 Hz artifact. Simulated artifact was removed using different averaged segments that varied by the number of periods per segment. The zoomed-in portion of each chart highlights a limitation in the available number of averaged segments in different conditions (e.g., varying number of periods in each segment), and highlights the plateau phase. Due to the observed plateau phase, SPD values were determined for a range of 10–100 averaged segments in increments of 2, and in the range of 100–2000 in increments of 50. The original signal was obtained from the O2 electrode from the first participant, and downsampled to 500 Hz. **(A)** Results of SPD in the alpha band after simulated filtration prepared on block *sh2* (i.e., eyes closed) and *sh5* (i.e., eyes open). SPD in the alpha band was counted in the range of 8–12 Hz. **(B)** Results of SPD in 10 Hz after the simulated filtration prepared on block *sh2* (i.e., eyes closed) and *sh5* (i.e., eyes open). SPD in 10 Hz counted in the range of 9.5–10.5 Hz. **(C,D)** Spectrum percentage difference (SPD) between the original signal and the resulting signal after the removal of simulated 10 Hz artifact. Simulated artifact was removed using different averaged segments that varied by the number of periods per segment. The original signal was obtained from the O2 electrode from the first participant and was downsampled to 500 Hz. SPD values were determined according to the relationship described in Formula (9). The artifact template was created by storing the selected fixed number of oscillations to create an artifact template (i.e., 1200, 900, 600, …). Then, for a given number of periods in each segment, the appropriate number of averaged segments was selected. **(C)** SPD values in the alpha band (i.e., 8–12 Hz). **(D)** SPD values in 10 Hz (9.5–10.5 Hz).

To show quantitative differences in the results more clearly, Figure 7 report SPD values (defined in “Materials and Methods,” section “Method for Selecting the Optimal Parameters of Filtration”). The percentage difference in alpha band power (see Figure 7A) between the primary and filtered signal depends on the number of averaged segments, and the number of periods in a given segment. To better illustrate the differences, the *x*-axis is shown on a logarithmic scale. Based on Figure 7A, the use of ten 1-period segments to calculate the artifact template for a 1-period segment results in an 80% change in alpha power as compared to the original signal recorded during the eyes closed condition. For the eyes open condition, there was a 60% alpha power change from the original signal. Increasing the number of 1-period segments used for averaging decreases these differences to the point at which the further extension of the averaging window does not significantly change the accuracy of removing the artifact (i.e., a plateau phase). In our opinion, the starting point of the plateau effect may indicate the most effective values of filtration parameters. Using multiperiod segments results in a similar relation, but the differences start a lower percentage value for short averaging windows (i.e., smaller than 300 segments). Due to the limited number of oscillations in the 5-min signal (i.e., 300 s * 10 oscillations in every second = 3,000) and the selected number of periods in one segment, a limited number of segments were available for averaging. If the signal contains 3,000 oscillations, its division into two-periods segments results in 1,500 segments that can be used for averaging. Similarly, the division into 10-period segments will result in a relatively small number of segments for SMA (300). Taken together, we observed a relatively low difference in the spectrum, averaging for example at 400 segments for 1, 2, 3, etc. periodic division. This observation allows us to conclude that increasing the number of periods in a given segment forces the averaging of a longer signal fragment to obtain a satisfactorily low difference between signals. As described in the “Materials and Methods” section, artifact may change over time due to several reasons, including progressive changes in the electric potential on the skin-electrode interface. It is therefore desirable to use the shortest possible portion of the signal with stimulation for averaging. It is also important to note that, as the number of periods per segment increases, the plateau effect becomes less apparent. For example, this effect can be seen in the zoomed-in charts shown in Figure 7 without the logarithmic scale on the *x*-axis. Such property makes it difficult to identify the optimal filtration parameters. The percent values of changes in the spectrum for 10 Hz (see Figure 7B) show a similar trend as observed for the alpha band. In particular, starting from 10 to 600 segments, the power difference between signals at 10 Hz decreased from 97 to 7% in the eyes closed condition and decreased from 95 to 6% in the eyes open condition. Further extension of the averaging window does not significantly improve the filtration accuracy, i.e., the observed changes are on the order of 0.1%. Given the observed changes to the original spectrum induced by our applied algorithm, it seems reasonable to select the filtration parameters using 1-period segments that take into account ~5% of the entire signal (i.e., 600 1-period segments from all 3000 oscillations).

In contrast to the above analyses, we checked for differences in the spectrum based on the length of signal taken for averaging (see Figures 7C,D). The approach was as follows: first, we selected the signal length [e.g., 1200 oscillations (120 s signal), 900 oscillations (90 s signal)]. Then, we determined the method for signal division (e.g., 1, 2, 3. periodic segments), and the SPD value was read for the number of averaged segments according to the following relationship:

(9) $$\begin{array}{l} period\;in\;one\;segment*averaged\;segments\\ =averaged\;oscillations \end{array}$$

The data shown in Figures 7C,D indicate that the smallest possible difference in the obtained spectrum decreases with an increasing length of the averaged signal used to create the artifact template. In the case of 1200 averaged oscillations (i.e., 40% of 5 min signal), the smallest difference in the alpha band stops at 3–4%. However, for a smaller number of averaged oscillations, this value increases by 4–5% for 900 and 600 oscillations, and by 10% for 300 oscillations. The differences increase by 14 and 24% for 200 and 100 oscillations, respectively (Figure 7C). This effect is even stronger for the SPD in the 10 Hz; in particular, the smallest difference results in a 31% increase for the condition with 100 oscillations (Figure 7D). It is also interesting to note that the number of periods in each segment has more influence on the SPD value when a smaller number of averaged oscillations is used. As the number of periods in each segment increases together with a smaller number of averaged oscillations, the observed differences in the spectrum increase much faster. This is particularly true for 300, 200, and 100 oscillations. These results are similarly observed for the analysis of differences in the spectrum of the signal with open eyes, and with low power in the alpha band. In order to more accurately illustrate the effects of frequency domain filtration, power spectra for various averaging parameters are provided in Supplementary Materials.

### Differences in the Time Domain

**FIGURE 8:**
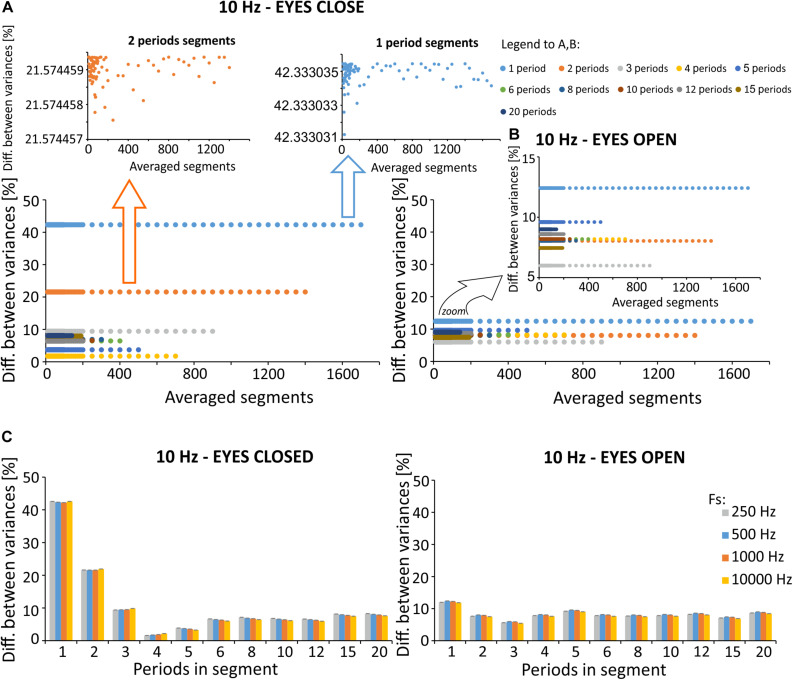
Percent difference between signal variances for the various exemplar selected simulation, calculated according to the relationship described in Formula (8). **(A)** Difference in variance for a varying number of periods per segment, prepared using signal with excitation in the alpha band (eyes closed). Zoomed-in displays for 1- and 2-period segments were prepared to improve visibility of relatively small differences that vary based on averaged segments for the selected number of periods per segment. **(B)** Difference in variance for various number of periods per segment prepared using signal with inactivity in the alpha band (eyes open). **(C)** Averaged differences for various number of periods determined for different sampling frequencies. The original signal was recorded with a sampling frequency of 10 000 Hz, and downsampled in factors of 10, 20, and 40. This allowed us to analyze the signal with a sampling frequency of 1000 Hz, 500 Hz, and 250 Hz.

We discovered changes (2–42%) in signal variation following SMA with different filtration parameters (see the 8th equation in the “Materials and Methods” section), as compared to the original signal variance (see Figures 8A,B). The eyes open condition is characterized by less pronounced changes in variance (6–13%) as compared to the eyes closed condition (2–42%). Of note, the effects of the number of periods per segment are larger than the number of average segments, when considering the difference in variance between the original and the filtered signals. Changes between signals with a varying number of periods per segment are significantly larger [condition: eyes closed, *p* < 0.001, χ^2^ = 544.465, df = (10, 539), Kruskal-Willis; condition: eyes open, *p* < 0.001, χ^2^ = 544.465, *df* = (10, 539), Kruskal-Willis] than changes induced by a varying number of averaged segments [condition: eyes closed, *p* = 1, χ^2^ = 0.371, *df* = (49, 500), Kruskal-Willis; condition: eyes open, *p* = 1, χ^2^ = 0.574, *df* = (49, 500), Kruskal-Willis].

Due to the small differences in variance observed for a selected number of periods per segment, we also evaluated the effects of sampling frequency on the observed results in the time domain (Figure 8C). Registrations were made with the original sampling rate, which was equal to 10,000 Hz. To assess the effects of lower frequencies on changes in variances following SMA filtration, the signal was downsampled appropriately to 1000, 500, and 250 Hz. The analysis failed to identify the most effective sampling frequency for the removal of 10 Hz artifact.

### Effects of SMA Filtering on the EEG Signal Recorded From Electrode F4

**FIGURE 9:**
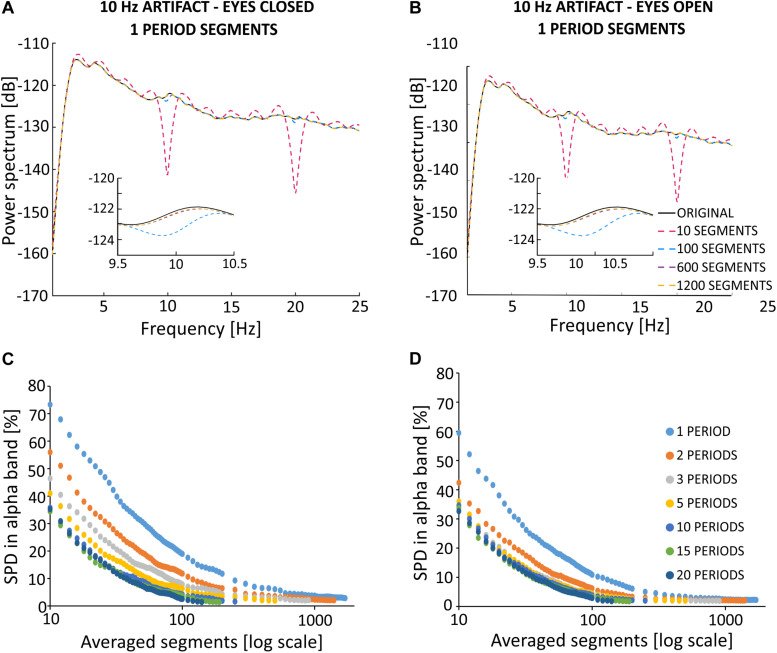
Effects of SMA filtration on EEG signal recorded from the F4 electrode with superimposed artificial stimulation artifact. The F4 electrode is situated closer to the stimulation electrodes than the O2 electrode. Thus, the amplitude of contamination by the stimulation artifact is higher for F4 as compared to O2. **(A)** Results of simulated filtration prepared on block *sh2* (eyes closed). **(B)** Results of simulated filtration prepared on block *sh5* (eyes open). Blocks *sh2* and *sh5* cause the sham registration in the conditions with eyes closed and eyes open. Spectrum percentage difference (SPD) between the original signal and the signal obtained after removal of the simulated 10 Hz artifact, which varies depending on the averaged segments for a varying number of periods per segment for the eyes closed **(C)** and eyes open **(D)** conditions.

As described in previous studies (Fehér and Morishima, 2016), the amplitude of the stimulation artifact is not the same on all recording electrodes. Indeed, the amplitude has been shown to depend on the distance between the stimulation and recording electrodes. Given this known association, we investigated the effects of SMA filtration on electrode F4, using similar simulation principles as applied above for O2. Compared to electrode O2, electrode F4 is characterized by a larger stimulation artifact amplitude. Thus, we added an artificial sinusoidal stimulation artifact to the uncontaminated EEG signal from the F4 electrode using an artificial sinusoid amplitude that is two times greater than the amplitude added to O2 (0.0004 V). Figure 9 presents the SMA filtration effects in the frequency domain for a varying number of averaged 1-period segments on the signal obtained during the eyes closed (Figure 9A) and eyes open conditions (Figure 9B). These signals were recorded from the F4 electrode and downsampled to 500 Hz. The obtained spectra appear to be similar to the spectra observed for the O2 electrode. For the 10 1-period segments applied to the signal from the eyes closed condition, we observed strong notches in the power spectrum occurring at 10 Hz and for its harmonics. The use of a larger number of averaged segments was associated with a gradual approximation of the amplitude of the signal after filtration to the amplitude of the primary signal. The use of 10 1-period segments to calculate the artifact template for a 1-period segment results in a signal that differs from the original one by 74% in alpha power for the eyes closed condition (Figure 9C) and by 60% for the eyes open condition (Figure 9D). Increasing the number of 1-period segments used for averaging decreases these differences to the point at which the further extension of the averaging window does not significantly change the accuracy artifact removal (i.e., plateau phase). A similar pattern was observed for the analysis of the O2 electrode, with a smaller stimulation artifact.

### Effects of SMA Filtration on EEG Signal With toACS Stimulation Artifact

**FIGURE 10:**
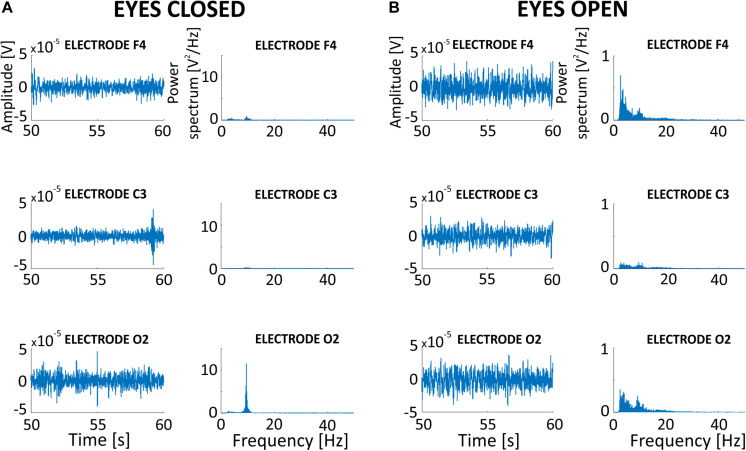
Effects of SMA filtration on EEG signal recorded during 10 Hz sinusoidal toACS from three different electrodes (F4, C3, and O2) during both eyes open **(A)** and eyes closed **(B)** conditions. EEG signals were filtered with a moving window that consisted of 600 1-period segments.

We investigated the impact of SMA on real signal recorded during toACS from three different electrodes (F4, C3, and O2) recorded during both eyes open (Figure 10A) and eyes closed (Figure 10B) conditions. The original 5-min signals recorded during real 10 Hz stimulation were filtered with an SMA window that contained 600 1-period segments. Power spectra obtained from different electrodes allowed us to conclude that SMA filtration is useful for the general observation of different brain regions. This was despite the presence of neural activity or inactivity in the alpha band in the case of ∼10 Hz stimulation frequency, or any other frequency band that was identical to the stimulation frequency.

## Discussion

Several studies report positive effects of different forms of ACS applied to the visual modality. In human studies of glaucoma or optic neuropathy, 10 days of toACS results in enhanced visual functions, enlarged visual fields, improved visual acuity, and decreased reaction time to visual stimuli, as well as improved vision-related quality of life (Fedorov et al., 2011; Gall et al., 2011; Sabel et al., 2011). A recent pilot clinical study shows similar effectiveness of long-term treatment (i.e., 4–6 years) with transcorneal(tco)ACS (Ota et al., 2018). Moreover, tcoACS has also proven to be useful in the treatment of retinitis pigmentosa, wherein recent clinical studies in humans demonstrate both structural and functional improvement (Schatz et al., 2011; Bittner and Seger, 2018; Bittner et al., 2018, however, see Wagner et al., 2017). Several other clinical studies demonstrate that tcoACS can improve visual function (i.e., visual acuity and/or visual field) in patients with various retinal diseases, including retinal artery occlusion, traumatic optic neuropathy, non-arteritic ischemic optic neuropathy, and Best vitelliform macular dystrophy (for a review, see Ota et al., 2018).

The mechanism of action of the various forms of ACS as a therapeutic method is still debated. Animal studies indicate that the therapeutic effects of toACS are due, in part, to neuroprotection of the retinal ganglion cells and decreased degeneration of photoreceptors (Hanif et al., 2016). Neuroprotective and pro-regenerative effects found in rodent models of optic neuropathies and retinal degeneration suggest that both toACS and tcoACS exert their effects via an upregulation of neurotrophic factors and a downregulation of pro-inflammatory pathways [for review, see Sehic et al., 2016; Antal et al., 2017 (submitted)]. Positive effects of toACS and tcoACS are not limited to the retina; indeed, the positive effects may also include changes in brain rhythms. Human and animal studies have shown that stimulation aftereffects also include modifications in neuronal oscillations (i.e., frequency, amplitude and phase), which are known as cortical entrainment (Sergeeva et al., 2015; Gall et al., 2016).

Considering the complexity of mentioned phenomena, there may be objections to whether examination of the acute effect can effectively help to understand the nature of processes related to toACS and tcoACS. There is a possibility, however, that therapeutic value of stimulation is related to brain plasticity. Despite this, observing brain activity during stimulation seems to be extremely helpful in understanding the mechanisms responsible for stable post-stimulation plastic changes.

The present study aimed to solve the problem of removing the EEG signal artifact that results from sinusoidal toACS. We designed an algorithm that allows the user to modify two parameters: (1) the number of sinusoidal periods present in each segment of the divided signal and (2) the number of segments (centered around the segment that included the artifact) used to calculate the artifact template for each segment. The selection of these parameters depends on the sampling frequency of the signal, the duration of the stimulation, and its frequency. Evaluation of the accuracy of artifact removal during real toACS is difficult due to the lack of relevant knowledge about the level to which brain oscillations are entrained by the electrical stimulus during simultaneous EEG recording. A reliable assessment of the accuracy of removing the stimulation artifact is currently possible only on simulation data, in which the information about the primary EEG signal is “hidden” under artificially superimposed sine wave representing sinusoidal toACS artifact. It is then possible to compare the original EEG signal with the EEG signal obtained after filtration in both time and frequency domains.

To choose the optimal parameter values of the SMA-based stimulation artifact removal, the approach we propose assumes that the correct pre-stimulation simulations are performed on the clean EEG signal (i.e., without real stimulation artifact). This approach involves applying a sinusoidal function to the known EEG signal (i.e., reference signal) that is similar in amplitude similar to the function that occurs during real stimulation. In addition, the frequency of the sinusoidal function should be the same as the frequency of the stimulation. The reference signal also should be the same length as the signal recorded during real stimulation and should reflect the condition that the subject was in during actual stimulation. For example, in a paradigm focused on alpha band activity excitation (via, for example, closing the eyes), the reference signal should also include an excitation in alpha band activity. The use of quantitative measures allows for the selection of appropriate filtering parameters, for example: (1) a difference in the power spectrum and/or (2) variance between the reference signal and the signal that resulted from the application of various filtering parameters.

When choosing the optimal averaging parameters, one should bear in mind the fact that in real stimulations, the electrical artifact changes progressively with time. Therefore, the length of the averaging window used to calculate the artifact template should be as short as possible, with minimal difference between the power of the reference signals and the filtered signals. As shown in Figure 7 for the power domain, the difference between the filtered and the original signal decreases with an increase in the length of the averaged signal. A similar observation has been reported in the removal of deep brain stimulation artifact using an algorithm for creating a stimulation artifact template (Sun and Hinrichs, 2016). Analysis of the accuracy of artifact removal on artificial stimulation data showed power differences in the alpha band between the primary and filtered signals that range from 80 to 2% for different averaging window lengths on signal collected during an eyes closed condition. These differences range from 60 to 2% on signal collected during an eyes open condition. The dynamics of SPD can be characterized exponentially as follows: (1) SPD values decrease with an increase in the signal length used for artifact template creation and (2) the slope of the exponential curve decreases as the number of periods per segment increases. Considering the optimal filtration parameters, it is important to take into account the occurrence of the plateau phase, which is a reflection of the phenomenon that a progressive increase in the number of averaged segments does not substantially improve the accuracy of artifact removal. We predicted a positive effect of averaging the shortest possible signal to remove the artifact from real signals. This prediction allows to conclude that the plateau phase is a basis for inferring the correct choice of length of the averaged signal that is used in the creation of the artifact template. Our conclusions are consistent with previous work that has examined challenges related to removal of sinusoidal artifact. In particular, our results confirm the appropriateness of selecting an averaging window equal to 2.5% of the length of the full signal with stimulation (Helfrich et al., 2014). The approach involved creating an artifact template for a 30-period segment based on 10 neighboring segments centered on the segment that included the target artifact. In the case described by Helfrich and colleagues which included a 10 Hz stimulation that lasted 20 min, this means that 300 out of the total 12,000 oscillations are used for the template (i.e., 2.5% of the entire signal length). In our analysis, a 2.5% averaging window results in the selection of 750 1-period segments from a total of 3000 oscillations. This choice results in an ~6% difference between the original and the filtered signal in the case of excitation or inactivity in the alpha band. The use of 2- and 3-period averages of 750 segments is in accordance with the results presented in Figure 7, and shows a more favorable difference between spectra. However, this approach requires using a larger portion of the signal. The second method proposed in the literature concerns the selection of a window that is 5% of the length of the entire signal and has the smallest possible number of oscillation periods. In our case, this approach would be an average of 150 1-period segments (Kohli and Casson, 2015). According to the simulation results, such averaging is associated with an error in the power spectrum in the alpha band that reaches almost 17% in the case of excitation (i.e., eyes closed) and 8% in the case of inactivity (i.e., eyes open). Under these parameters, the peak amplitude at 10 Hz reaches 22% in both states of activity. Due to the inability to observe the plateau phase for multiperiod data and under short stimulation times, the use of 1-period segments shows a clear plateau phase even in 5-min of signal.

Analysis of differences in the variance between the primary signal and signal after filtration showed that the SMA method induced large percentage changes. In particular, filtering the signal with increased power in the alpha band is characterized by a large percentage difference (42%) for the 1-period segment. This is a prerequisite for using SMA for signal analysis, primarily in the frequency domain. However, it is clear from the spectra obtained after SMA filtration that the algorithm partially removes the stimulation frequency from the spectrum. Adequate maneuvering with averaging parameters minimizes the removal effect.

Recently, an alternative solution has been proposed (Witkowski et al., 2016) to address the problem that the stimulation artifact obscures brain activity in the frequency range of interest: amplitude-modulated ACS (AM-ACS). This new method is based on stimulation with a specific wave created from two frequencies: (1) a high carrier frequency that is not related to brain oscillation and (2) a modulated low target frequency. The AM-ACS signal spectrum in the frequency range of interest is devoid of the large artifact observed during traditional non-modulated ACS. Unfortunately, although this is a relatively new and promising electrical stimulation paradigm, AM-ACS requires additional research to provide answers to many related issues. For example, a recent study compared the effects of ACS and AM-ACS on simulation data with modeled visual cortex (Negahbani et al., 2018). Results of this study indicate that, in the AM-ACS method, it is necessary to use a significantly larger amplitude of stimulation than in traditional ACS to observe similar effects in cortical activity. To the best of our knowledge, no studies using real EEG signals exist, which could prove in detail that AM-ACS and traditional ACS have the same effects on brain activity. Such evidence would be required for researchers to shift toward AM-ACS without negative effects and would circumvent the problems associated with the ACS artifact. The algorithm we propose here for removing the sinusoidal toACS artifact may improve the accuracy of analyses using signals acquired during ACS and AM-ACS stimulation. This algorithm would allow one to verify and compare the usefulness and imperfections of both methods.

Considering the applicability of the proposed method, it is also worth paying attention to the alternative fMRI research option proposed in a number of papers (Vosskuhl et al., 2016; Chai et al., 2018; Kar et al., 2020). Compared to fMRI, EEG is commonly used in clinical practice and has a very good temporal resolution allowing observations of changes in the brain related to numerous deficits and diseases. Lower availability and much higher cost of the fMRI cannot completely replace the EEG-based research and its usefulness in clinics.

One unquestionable advantage of SMA filtration is the simplicity of the algorithm implementation, and presence of a clear operating principle. The two main parameters of the algorithm directly translate into periodic signal specificity with sinusoidal ACS. It is also important to note that the use of SMA filtration is associated with the assumption that the noise to be removed is additive. Previous research suggests the non-linear nature of the unwanted component in the signal, which may be a limitation to the applicability of the averaging algorithm to remove the ACS artifact (Noury et al., 2016). In response to these reports, Neuling et al. (2017) provided rationale for observing the non-linearity of the ACS artifact, which involved not exceeding the limits of technical stimulators. For this reason, in the present study we carefully monitored the impedance of the electrodes to ensure that the sidebands described in this study are not affected. Due to the aforementioned limitations in the use of SMA resulting from the non-linearity of the artifact caused by the hardware settings, the effect of SMA filtration on the signal from a different recording system was used in the main body of the article. These results are reported in Supplementary Materials. The analysis performed on the second signal did not reveal any changes in the effects of SMA filtration that significantly differ from the conclusions described herein. Despite mentioned discussion between research group, no final arrangements have been made about to what extent the artifact should be removed for the signal to be useful. There is a possibility that the proposed cleaning technique can remove some of the recorded ongoing neural activity. Due to this, we prepared detailed analysis in time and frequency domain for simulated data where we have 100% knowledge about original EEG information before adding stimulation artifact. This approach allows us to extract percentage value of accuracy of removing the artifact, which could be useful in planning protocol of stimulation and preprocessing step in EEG with simultaneous stimulation analysis.

## Data Availability Statement

The datasets generated for this study are available on request to the corresponding author.

## Ethics Statement

The studies involving human participants were reviewed and approved by Ethical Committee of the University of Warsaw. The patients/participants provided their written informed consent to participate in this study.

## Author Contributions

MŻ: design and implementation of the filtration method in the Matlab environment and data analysis. MŻ, PD, and WW: writing and reviewing of the manuscript. PD and WW: study design. PD: data acquisition and EEG data pre-processing. WW: supervision. All authors contributed to the article and approved the submitted version.

## Conflict of Interest

The authors declare that the research was conducted in the absence of any commercial or financial relationships that could be construed as a potential conflict of interest.

## References

[B1] Allen P. J. Josephs O. Turner R. (2000). A method for removing imaging artifact from continuous EEG recorded during functional MRI. *Neuroimage* 12 230–239. 10.1006/nimg.2000.0599 10913328

[B2] Antal A. Alekseichuk I. Bikson M. Brockmöller J. Brunoni A. R. Chen R. (2017). Low intensity transcranial electric stimulation: safety, ethical, legal regulatory and application guidelines. *Clin. Neurophysiol.* 128 1774–1809. 10.1016/j.clinph.2017.06.001 28709880 PMC5985830

[B3] Bénar C.-G. Aghakhani Y. Wang Y. Izenberg A. Al-Asmi A. Dubeau F. (2003). Quality of EEG in simultaneous EEG-fMRI for epilepsy. *Clin. Neurophysiol.* 114 569–580. 10.1016/S1388-2457(02)00383-8 12705438

[B4] Bittner A. K. Seger K. (2018). Longevity of visual improvements following transcorneal electrical stimulation and efficacy of retreatment in three individuals with retinitis pigmentosa. *Graefes Arch. Clin. Exp. Ophthalmol.* 256 299–306. 10.1007/s00417-017-3858-8 29222719 PMC6039224

[B5] Bittner A. K. Seger K. Salveson R. Kayser S. Morrison N. Vargas P. (2018). Randomized controlled trial of electro-stimulation therapies to modulate retinal blood flow and visual function in retinitis pigmentosa. *Acta Ophthalmol.* 96 e366–e376. 10.1111/aos.13581 29130647 PMC5920686

[B6] Brignani D. Ruzzoli M. Mauri P. Miniussi C. (2013). Is transcranial alternating current stimulation effective in modulating brain oscillations? *PLoS One* 8:e56589. 10.1371/journal.pone.0056589 23457586 PMC3573000

[B7] Chai Y. Sheng J. Bandettini P. A. Gao J.-H. (2018). Frequency-dependent tACS modulation of BOLD signal during rhythmic visual stimulation. *Hum. Brain Mapp.* 39 2111–2120. 10.1002/hbm.23990 29389051 PMC6141005

[B8] Dowsett J. Herrmann C. S. (2016). Transcranial alternating current stimulation with sawtooth waves: simultaneous stimulation and EEG recording. *Front. Hum. Neurosci.* 10:135. 10.3389/fnhum.2016.00135 27065835 PMC4809871

[B9] Fedorov A. Jobke S. Bersnev V. Chibisova A. Chibisova Y. Gall C. (2011). Restoration of vision after optic nerve lesions with noninvasive transorbital alternating current stimulation: a clinical observational study. *Brain Stimul.* 4 189–201. 10.1016/j.brs.2011.07.007 21981854

[B10] Fehér K. D. Morishima Y. (2016). Concurrent electroencephalography recording during transcranial alternating current stimulation (tACS). *J. Vis. Exp.* 22:e53527. 10.3791/53527 26862814 PMC4828151

[B11] Gall C. Schmidt S. Schittkowski M. P. Antal A. Ambrus G. G. Paulus W. (2016). Alternating current stimulation for vision restoration after optic nerve damage: a randomized clinical trial. *PLoS One* 11:e0156134. 10.1371/journal.pone.0156134 27355577 PMC4927182

[B12] Gall C. Sgorzaly S. Schmidt S. Brandt S. Fedorov A. Sabel B. A. (2011). Noninvasive transorbital alternating current stimulation improves subjective visual functioning and vision-related quality of life in optic neuropathy. *Brain Stimul.* 4 175–188. 10.1016/j.brs.2011.07.003 21981853

[B13] Gramfort A. Luessi M. Larson E. Engemann D. A. Strohmeier D. Brodbeck C. (2013). MEG and EEG data analysis with MNE-Python. *Front. Neurosci.* 7:267. 10.3389/fnins.2013.00267 24431986 PMC3872725

[B14] Hanif A. M. Kim M. K. Thomas J. G. Ciavatta V. T. Chrenek M. Hetling J. R. (2016). Whole-eye electrical stimulation therapy preserves visual function and structure in P23H-1 rats. *Exp. Eye Res.* 149 75–83. 10.1016/j.exer.2016.06.010 27327393 PMC4985439

[B15] Helfrich R. F. Schneider T. R. Rach S. Trautmann-Lengsfeld S. A. Engel A. K. Herrmann C. S. (2014). Entrainment of brain oscillations by transcranial alternating current stimulation. *Curr. Biol.* 24 333–339. 10.1016/j.cub.2013.12.041 24461998

[B16] Jas M. Larson E. Engemann D. A. Leppäkangas L. Taulu S. Hämäläinen M. (2018). A reproducible MEG/EEG group study with the MNE software: recommendations, quality assessments, and good practices. *Front. Neurosci.* 12:530. 10.3389/fnins.2018.00530 30127712 PMC6088222

[B17] Kanai R. Chaieb L. Antal A. Walsh V. Paulus W. (2008). Frequency-dependent electrical stimulation of the visual cortex. *Curr. Biol.* 18 1839–1843. 10.1016/j.cub.2008.10.027 19026538

[B18] Kar K. Ito T. Cole M. W. Krekelberg B. (2020). Transcranial alternating current stimulation attenuates BOLD adaptation, and increases functional connectivity. *J. Neurophysiol.* 123 428–438. 10.1152/jn.00376.2019 31825706 PMC6985864

[B19] Kasten F. H. Dowsett J. Herrmann C. S. (2016). Sustained Aftereffect of α-tACS Lasts Up to 70 min after stimulation. *Front. Hum. Neurosci.* 10:245. 10.3389/fnhum.2016.00245 27252642 PMC4879138

[B20] Kohli S. Casson A. J. (2015). “Removal of Transcranial a.c. Current Stimulation artifact from simultaneous EEG recordings by superposition of moving averages,” in *Proceedings of the 2015 37th Annual International Conference of the IEEE Engineering in Medicine and Biology Society (EMBC). Presented at the 2015 37th Annual International Conference of the IEEE Engineering in Medicine and Biology Society (EMBC)* (Milan: IEEE), 3436–3439. 10.1109/EMBC.2015.7319131 26737031

[B21] Kohli S. Casson A. J. (2019). Removal of gross artifacts of transcranial alternating current stimulation in simultaneous EEG monitoring. *Sensors* 19:190. 10.3390/s19010190PMC6338981 30621077 PMC6338981

[B22] Moreno-Duarte I. Gebodh N. Schestatsky P. Guleyupoglu B. Reato D. Bikson M. (2014). “Transcranial Electrical Stimulation: transcranial Direct Current Stimulation (tDCS), Transcranial Alternating Current Stimulation (tACS), Transcranial Pulsed Current Stimulation (tPCS), and Transcranial Random Noise Stimulation (tRNS),” in *The Stimulated Brain*, Ed. Kadosh R. C. (Amsterdm: Elsevier), 35–59. 10.1016/B978-0-12-404704-4.00002-8

[B23] Negahbani E. Kasten F. H. Herrmann C. S. Fröhlich F. (2018). Targeting alpha-band oscillations in a cortical model with amplitude-modulated high-frequency transcranial electric stimulation. *Neuroimage* 173 3–12. 10.1016/j.neuroimage.2018.02.005 29427848 PMC5911251

[B24] Neuling T. Rach S. Herrmann C. S. (2013). Orchestrating neuronal networks: sustained after-effects of transcranial alternating current stimulation depend upon brain states. *Front. Hum. Neurosci.* 7:161. 10.3389/fnhum.2013.00161 23641206 PMC3639376

[B25] Neuling T. Ruhnau P. Weisz N. Herrmann C. S. Demarchi G. (2017). Faith and oscillations recovered: on analyzing EEG/MEG signals during tACS. *Neuroimage* 147 960–963. 10.1016/j.neuroimage.2016.11.022 27888060

[B26] Niazy R. K. Beckmann C. F. Iannetti G. D. Brady J. M. Smith S. M. (2005). Removal of FMRI environment artifacts from EEG data using optimal basis sets. *Neuroimage* 28 720–737. 10.1016/j.neuroimage.2005.06.067 16150610

[B27] Noury N. Hipp J. F. Siegel M. (2016). Physiological processes non-linearly affect electrophysiological recordings during transcranial electric stimulation. *Neuroimage* 140 99–109. 10.1016/j.neuroimage.2016.03.065 27039705

[B28] Ota Y. Ozeki N. Yuki K. Shiba D. Kimura I. Tsunoda K. (2018). The efficacy of transcorneal electrical stimulation for the treatment of primary open-angle glaucoma: a pilot study. *Keio J. Med.* 67 45–53. 10.2302/kjm.2017-0015-OA 29415904

[B29] Rompelman O. Ros H. H. (1986). Coherent averaging technique: a tutorial review. Part 1: noise reduction and the equivalent filter. *J. Biomed. Eng.* 8 24–29. 10.1016/0141-5425(86)90026-9 3951206

[B30] Sabel B. A. Fedorov A. B. Naue N. Borrmann A. Herrmann C. Gall C. (2011). Non-invasive alternating current stimulation improves vision in optic neuropathy. *Restor. Neurol. Neurosci.* 29 493–505. 10.3233/RNN-2011-0624 22124039

[B31] Sabel B. A. Hamid A. I. A. Borrmann C. Speck O. Antal A. (2019). Transorbital alternating current stimulation modifies BOLD activity in healthy subjects and in a stroke patient with hemianopia: a 7 Tesla fMRI feasibility study. *Int. J. Psychophysiol.* 154 80–92. 10.1016/j.ijpsycho.2019.04.002 30978369

[B32] Schatz A. Röck T. Naycheva L. Willmann G. Wilhelm B. Peters T. (2011). Transcorneal electrical stimulation for patients with retinitis pigmentosa: a prospective, randomized, sham-controlled exploratory study. *Invest. Ophthalmol. Vis. Sci.* 52 4485–4496. 10.1167/iovs.10-6932 21467183

[B33] Sehic A. Guo S. Cho K. S. Corraya R. M. Chen D. F. Utheim T. P. (2016). Electrical stimulation as a means for improving vision. *Am. J. Pathol.* 186 2783–2797. 10.1016/j.ajpath.2016.07.017 27643530 PMC5225285

[B34] Sergeeva E. G. Henrich-Noack P. Gorkin A. G. Sabel B. A. (2015). Preclinical model of transcorneal alternating current stimulation in freely moving rats. *Restor. Neurol. Neurosci.* 33 761–769. 10.3233/RNN-150513 25813371

[B35] Sun L. Hinrichs H. (2016). Moving average template subtraction to remove stimulation artefacts in EEGs and LFPs recorded during deep brain stimulation. *J. Neurosci. Methods* 266 126–136. 10.1016/j.jneumeth.2016.03.020 27039973

[B36] Vossen A. Gross J. Thut G. (2015). Alpha Power Increase After Transcranial Alternating Current Stimulation at Alpha Frequency (α-tACS) Reflects Plastic Changes Rather Than Entrainment. *Brain Stimulat.* 8 499–508. 10.1016/j.brs.2014.12.004 25648377 PMC4464304

[B37] Vosskuhl J. Huster R. J. Herrmann C. S. (2016). BOLD signal effects of transcranial alternating current stimulation (tACS) in the alpha range: a concurrent tACS–fMRI study. *Neuroimage* 140 118–125. 10.1016/j.neuroimage.2015.10.003 26458516

[B38] Wagner S. K. Jolly J. K. Pefkianaki M. Gekeler F. Webster A. R. Downes S. M. (2017). Transcorneal electrical stimulation for the treatment of retinitis pigmentosa: results from the TESOLAUK trial. *BMJ Open Ophthalmol.* 2:e000096. 10.1136/bmjophth-2017-000096 29354722 PMC5751865

[B39] Witkowski M. Garcia-Cossio E. Chander B. S. Braun C. Birbaumer N. Robinson S. E. (2016). Mapping entrained brain oscillations during transcranial alternating current stimulation (tACS). *Neuroimage* 140 89–98. 10.1016/j.neuroimage.2015.10.024 26481671

