# Supplementary material for: Removal of the Sinusoidal Transorbital Alternating Current Stimulation Artifact From Simultaneous EEG Recordings: Effects of Simple Moving Average Parameters
Source: Front Neurosci. 2020 Jul 29;14:735. doi: 10.3389/fnins.2020.00735 (PMC7403449; doi:10.3389/fnins.2020.00735)

Words: 1339

SUPPLEMENTARY MATERIALS:

SUPPLEMENTARY POWER SPECTRA:

To elucidate the optimal filtration parameters for the 10 Hz sinusoidal ACS artifact removal, we compared the original signal with the signal obtained after filtration using various SMA settings. Supplementary Figure 1 shows the effects of SMA filtration using a different number of averaged 1-period segments in the frequency domain. In particular, signal is shown for eyes closed (Figure 1A) and eyes open (Figure 1B) conditions, and recorded from the O2 electrode downsampled to 500 Hz (recorded from the male participant). The case of 10 single-period segments on signal with eyes closed is characterized by strong notches in the power spectrum occurring at 10 Hz and for its harmonics. The amplitude of the signal power in 10 Hz after this filtration (-126 dB) differs from the original amplitude (-106.1 dB) by almost 20 dB. Using a larger number of averaged segments is associated with a gradual approximation of the amplitude of the signal after filtration to the amplitude of the primary signal. Amplitudes of signals for 10 Hz after averaging windows with 100, 600, and 1200 segments differ from the original by 0.9 dB, 0.2 dB, and 0.1 dB, respectively. Analogous changes are observed in the eyes open condition. Indeed, selection of a 10-segment averaging window also results in strong notches for 10 Hz and its harmonics. The difference between the filtered and the original signal decreases as the averaging window (i.e., number of segments) extends.

Based on the data presented in Supplementary Figure 1, we reviewed the changes in the spectrum for filtration using multi-period segments (i.e., 2-, 3-, and 10-period segments) (see Supplementary Figure 2). Two- (Figure 2A) and three-period (Figure 2B) segments in the condition with the 10 segment averaged window results in more frequent notching in the power spectrum (in both the eyes closed and eyes open signal) as compared to the condition with 1-period segments (presented in Supplementary Figure 1). The notches after 2-period filtering are characterized by a greater amplitude than notches after 3-period filtering, for both the eyes open and eyes closed conditions. The difference in amplitude between the original and the filtered signal for 10 Hz decreases with an increasing number of averaged segments. This relationship is similar to the results from averaging the 1-period segments (Supplementary Figure 1). In the case of filtration with 10-period segments (Figure 2C), no notches are observed. This may be due to the fact that selecting 10 such segments involves averaging the signal with a considerable length of 10 s. In addition, as the number of periods in a given segment increases, the number of all available averaging segments is simultaneously reduced. For this reason, the subsequent graphs (Figure 2A, 2B, and 2C) show also different cases of selecting the number of averaged segments. For example, the charts in Figure 2C present filtration with 10-period segments. Indeed, results are shown for 10, 50, 100, and 150 segments from 300 available segments in 5 minutes of signal with 10 Hz stimulation. As we can observe in the spectral domain, the accuracy of removing the artifact with the SMA method depends on the applied averaging parameters.

SUPPLEMENTARY EFFECTS OF SMA ON REMOVAL OF SIMULATED ARTIFACTS AT 5 Hz AND 15 Hz:

To better understand the performance of SMA, we also evaluated the accuracy of SMA on the removal of simulated artifacts at 5 and 15 Hz. The simulation was prepared using similar steps as described above for 10 Hz artifact. For 5 Hz stimulation, we used 1500 1-period segments for the 5-minute block of the original signal. For the 15 Hz stimulation, in contrast,

we set 4500 segments per period, and used the same signal length. The SPD analysis is shown in Supplementary Figure 3A and B. Similar to what was observed for removing the 10 Hz artifact (compare with Supplementary Figure 1), we found that the phase plateau appears in a similar number of averaged segments. The number of averaged oscillations has a similar effect in creating differences between spectra, as demonstrated in the removal of the 10 Hz artifact. The use of multiperiod segments to create the artifact template from 1200 sinusoidal oscillations is characterized by similar SPD differences in almost every condition, and also in the condition with 1-period segments (see Supplementary Figure 4A and B).

SUPPLEMENTAR EXPERIMENTAL PROCEDURE:

Data from registration was carried out using EEGDigiTrack ExG-32-USB (32-channel ExG Amplifier). Eleven recording electrodes were set according to the 10-20 System (Fp1, Fp2, F3, F4, C3, C4, P3, P4, O1, O2, and Cz). Alternating sinusoidal current stimulation (amplitude 20 μA, frequency 10 Hz) was applied via four transorbital electrodes. It was one session with one stimulation applied for 10 min. The signal was recorded with sampling rate of 500 Hz. EEG electrodes were referenced to two connected ear electrodes (A1 and A2). At the beginning of analysis data was band-pass filtered with a cut-off frequencies of 0.5 and 70 Hz and also free from electric artifact 50 Hz by a band-stop filter.

The experimental procedure was divided into six 5 min blocks of recording with either the eyes open or closed (Supplementary Figure 5).

SUPPLEMENTARY EXPERIMENTAL RESULTS:

As in analysis reported in main article, the comparison in frequency domain for different parameters of filtration was prepared (Supplementary Figure 6). Using SMA algorithm to remove 10 Hz artifact from signal recorded during supplementary experiment results with similar effects as in Supplementary Figure 2. Averaging with 10 segments length window is insufficient and it results with strong notches characteristic for chosen number of periods in one segment. Gradual extension of the averaging window (100, 600 1200 segments) allows to observing reduction of the difference in the amplitude for a 10 Hz peak between the original and filtered signals. It is also noticed that using 10 periods in one segment results with absence of notches what is related with full number of averaging oscillation connected with number of periods in one segment and number of averaged segments.

Supplementary Figure 7 reports spectrum differences in alpha band (8-12 Hz) and 10 Hz (9.5 - 10.5 Hz) between original signal from electrode O2 and signal obtained after different filtration parameters. In comparison to charts from Figure 7 in main article the occurrence of the plateau phase is similar. Dynamic of changes SPD values with increasing number of averaged segments allow to observe plateau phase especially for small number of periods in one segment (1, 2, 3). The difference in the effect of the SMA filter on the signals from two different stimulations concerns specific values. In condition from Supplementary Figure 7 power difference are in range smaller than 70 %, especially values in alpha band are smaller than in mentioned Figure 7.

In comparison to the above analysis, the differences in spectrum (Supplementary Figure 8) has been checked for the length of the signal taken to averaging (as in 9th equal in article). As in previous analogical Figure 7C and 7D from original article there is observed relation about the smallest available differences with the longest part of averaging signal. This mainly applies to differences for the 10 Hz peak. An influence of chosen number of periods in

one segment is less important in long averaging segments (1200, 900, 600 segments) than in shorter windows. In mentioned shorter conditions the difference (100, 200 segments) in spectrum arises faster with increase number of periods in one segment.

The comparison in time domain for different parameters of filtration was prepared in Supplementary Figure 9. It is still observed that number of periods in one segment is the main factor in producing percentage differences between variances original and after filtration. In condition of selected number of periods in segment lengthening the averaging window doesn't change differences between variances.

Supplementary Figure 10 presents differences between spectrum of original and filtered signal after removing 5 Hz and 15 Hz artifact. It is also observed plateau phase which corresponds to the reconstruction of the spectrum closest to the original one without improving the result along with the extension of the averaging window.

SUPPLEMENTARY FIGURES LEGENDS

Supplementary Figure 1. Power domain analysis of the SMA 1-period filtration for a different number of averaged segments used to create the artifact template. The original signal was recorded from the O2 electrode from the first participant and was downsampled to 500 Hz. Each row of the figure provides a zoomed-in view of the range of the spectrum of interest. The top row allows for the evaluation of preprocessing effects that include removal of the 50 Hz artifact and its harmonics. **(A)** Results of simulated filtration prepared on block *sh2* (i.e., eyes closed). **(B)** Results of simulated filtration prepared on block *sh5* (eyes open).

Supplementary Figure 2. Power domain analysis of effect of SMA multi-period filtration for varying numbers of averaged segments. The original signal was obtained from the O2 electrode and downsampled to 500 Hz. Results of simulated filtration prepared on block *sh2* (eyes closed) and *sh5* (eyes open) for the following filtration conditions: 2-period segments **(A)**, 3-period segments **(B)** or 10-period segments **(C)**.

Supplementary Figure 3. Spectrum percentage difference (SPD) between the original signal and the obtained after removing simulated 5 Hz and 15 Hz artifact. The SPD depends on averaged segments for a varying number of periods per segment. Simulation was prepared on signal recorded from electrode O2 from the first participant during block *sh2* (i.e., eyes closed) and block *sh5* (i.e., eyes open). **(A)** SPD in 5 Hz was calculated in the 4.5-5.5 Hz range. **(B)** SPD was calculated for 15 Hz from the 14.5-15.5 Hz range.

Supplementary Figure 4. Spectrum percentage difference (SPD) between the original signal and the signal obtained after removing the simulated artifact at 5 Hz **(A)** and 15 Hz **(B)**. SPD varies based on the averaged segments for a varying number of periods per segment. SPD values were calculated according to the relationship described in Formula (9).

Supplementary Figure 5. Experimental procedure. The whole experiment lasted 30 min. during which the two middle 5-min. blocks st3 and st5 contained 10 Hz toACS stimulation (amplitude 20 µA). During blocks *st1*, *st3* and *st5* female volunteer has got eyes open and during block *st2*, *st4* and *st6* eyes close (grey squares).

Supplementary Figure 6. Power domain analysis of effect of SMA multi-period filtration different number of averaged segments. The original signal derive from O2 electrode with

500 Hz sampling frequency. **(A)** Results of simulated filtration prepared on block *st2* (eyes close). **(B)** Results of filtration on signal from *st1* block (eyes open). Every row presents 1, 2, 3 or 10-period condition of filtration.

Supplementary Figure 7. Spectrum differences (SPD) between original signal and signal obtained after different parameters of SMA filtration. Zoom charts shows ending parts of different periods curves, which is caused by limited number of available segments for different number of periods. X-axis with logarithmic scale was used to presents differences in SPD values in low number of averaged segments for chosen number of periods. **(A)** SPD in alpha band was counted from range 8-12 Hz. **(B)** SPD in 10 Hz was counted from 9.5 -10.5 Hz.

Supplementary Figure 8. Spectrum percentage differences (SPD) in **(A)** alpha band (8 - 12 Hz) and **(B)** 10 Hz on signal from conditions: eyes open and eyes close. SPD values were determined according to relationship (9).

Supplementary Figure 9. Percentage difference between signal variances counted as in equal (8). **(A)** Difference between variances for various number of periods in one segment prepared on signal with excitation in the alpha band (eyes close). **(B)** Difference between variances for various number of periods in one segment prepared on signal with inactivity in the alpha band (eyes open).

Supplementary Figure 10. Spectrum percentage difference (SPD) between the original signal and obtained after removing simulated **(A)** 5 Hz and **(B)** 15 Hz artifact, depending on averaged segments for various number of periods in one segments. Simulation prepared on signal from electrode O2 on block *st2* (eyes close) and block *st1* (eyes open). SPD in 5 Hz was determined from range 4.5 – 5.5 Hz, SPD in 15 Hz from range 14.5 Hz – 15.5 Hz.

Supplementary Figure 1

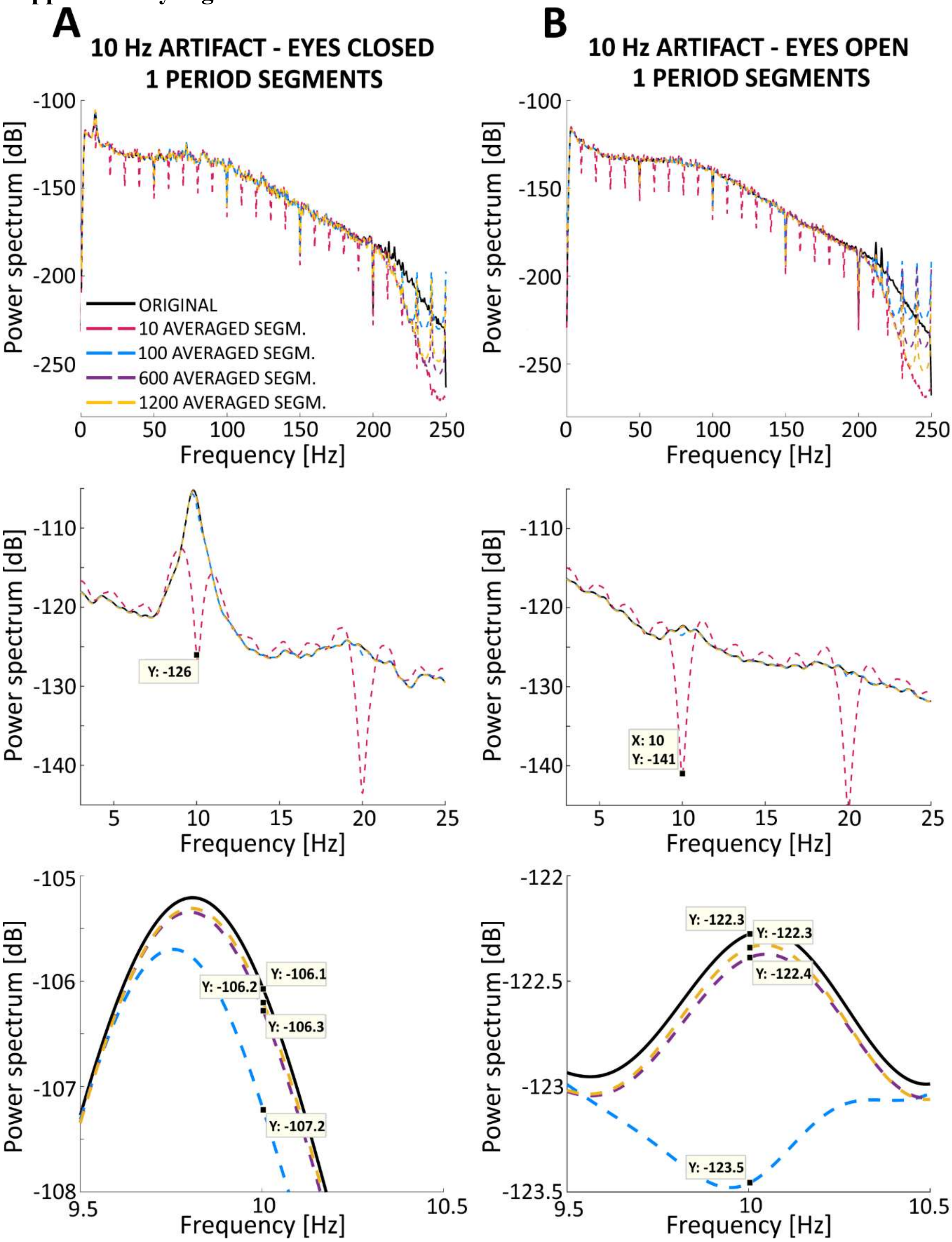

Supplementary Figure 2

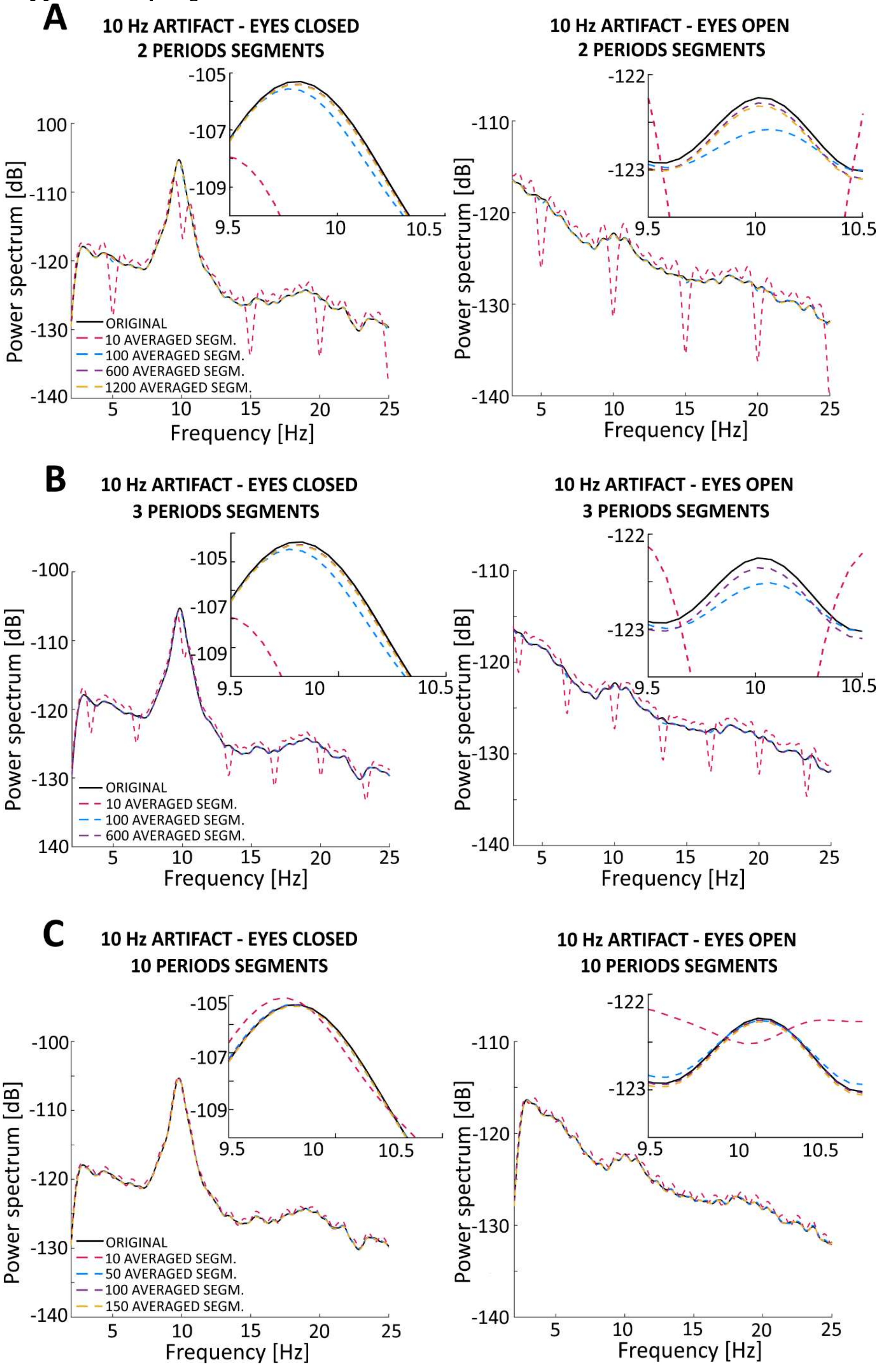

Supplementary Figure 3

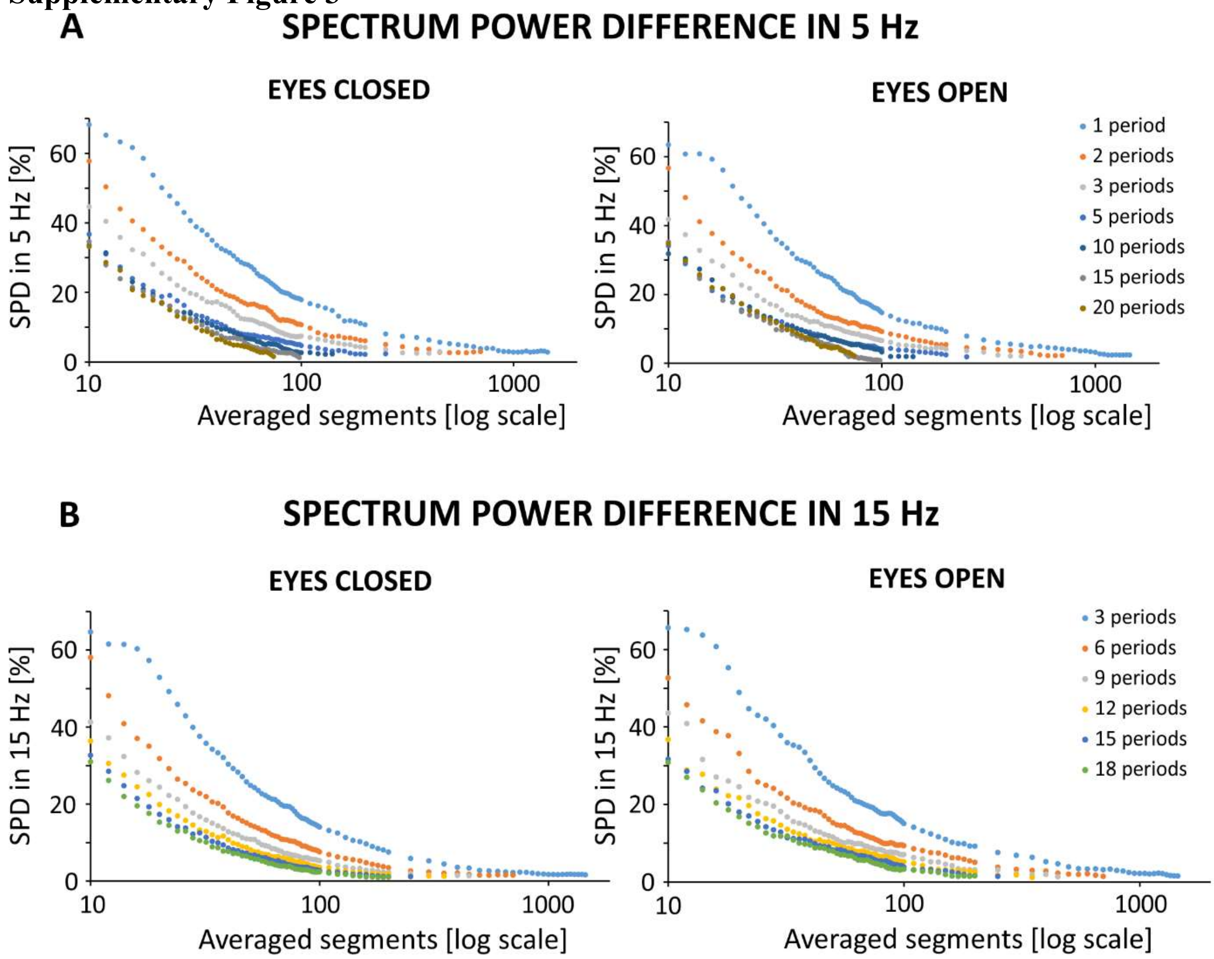

Supplementary Figure 4

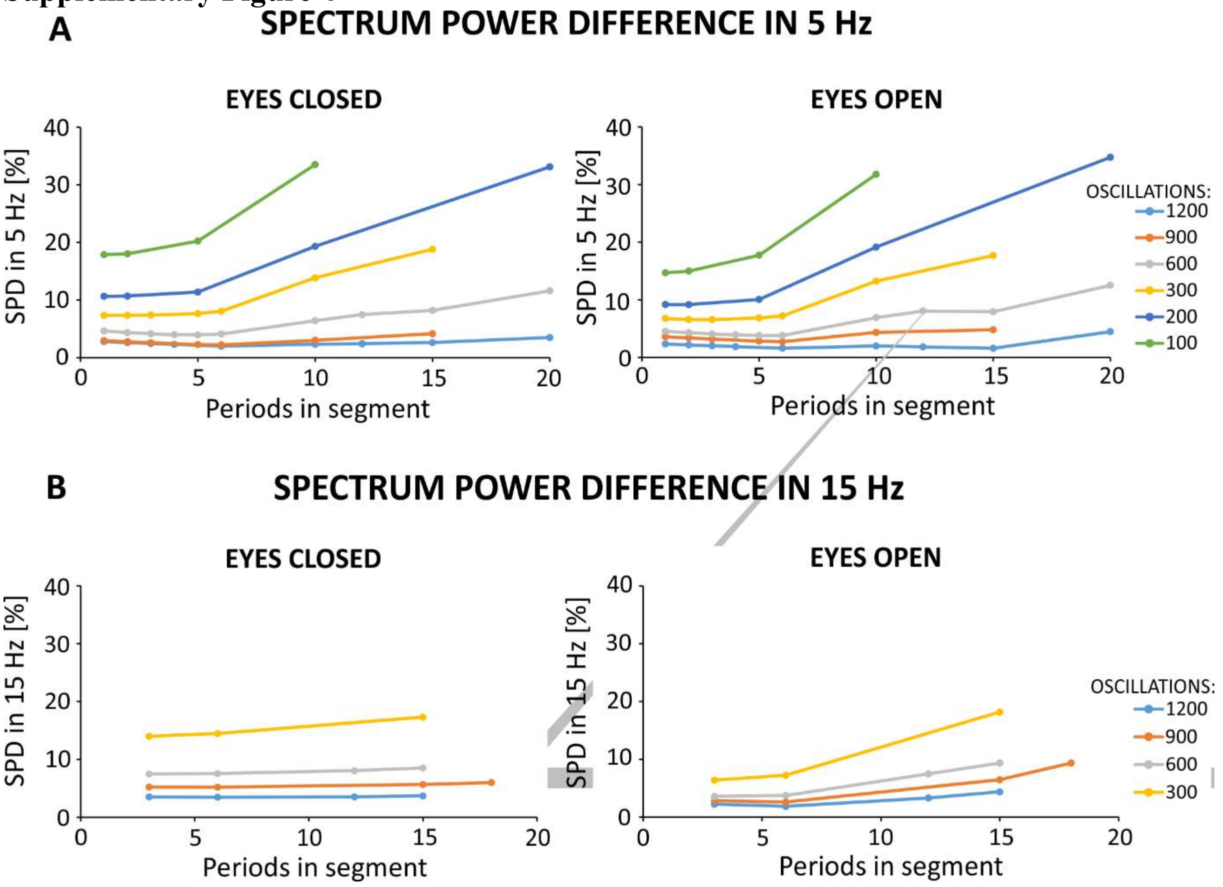

Supplementary Figure 5

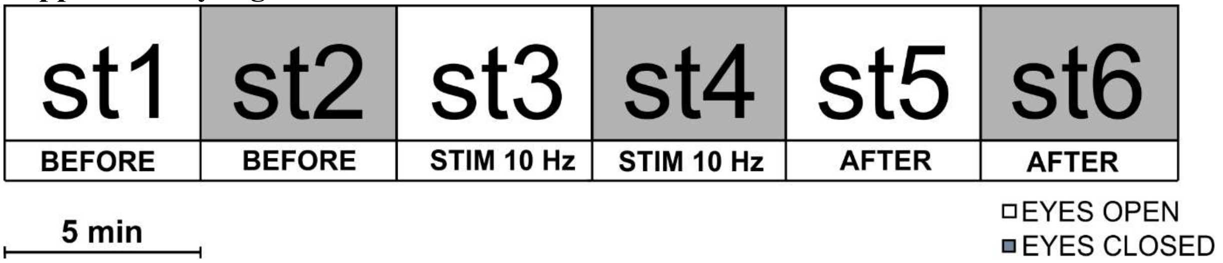

Supplementary Figure 6

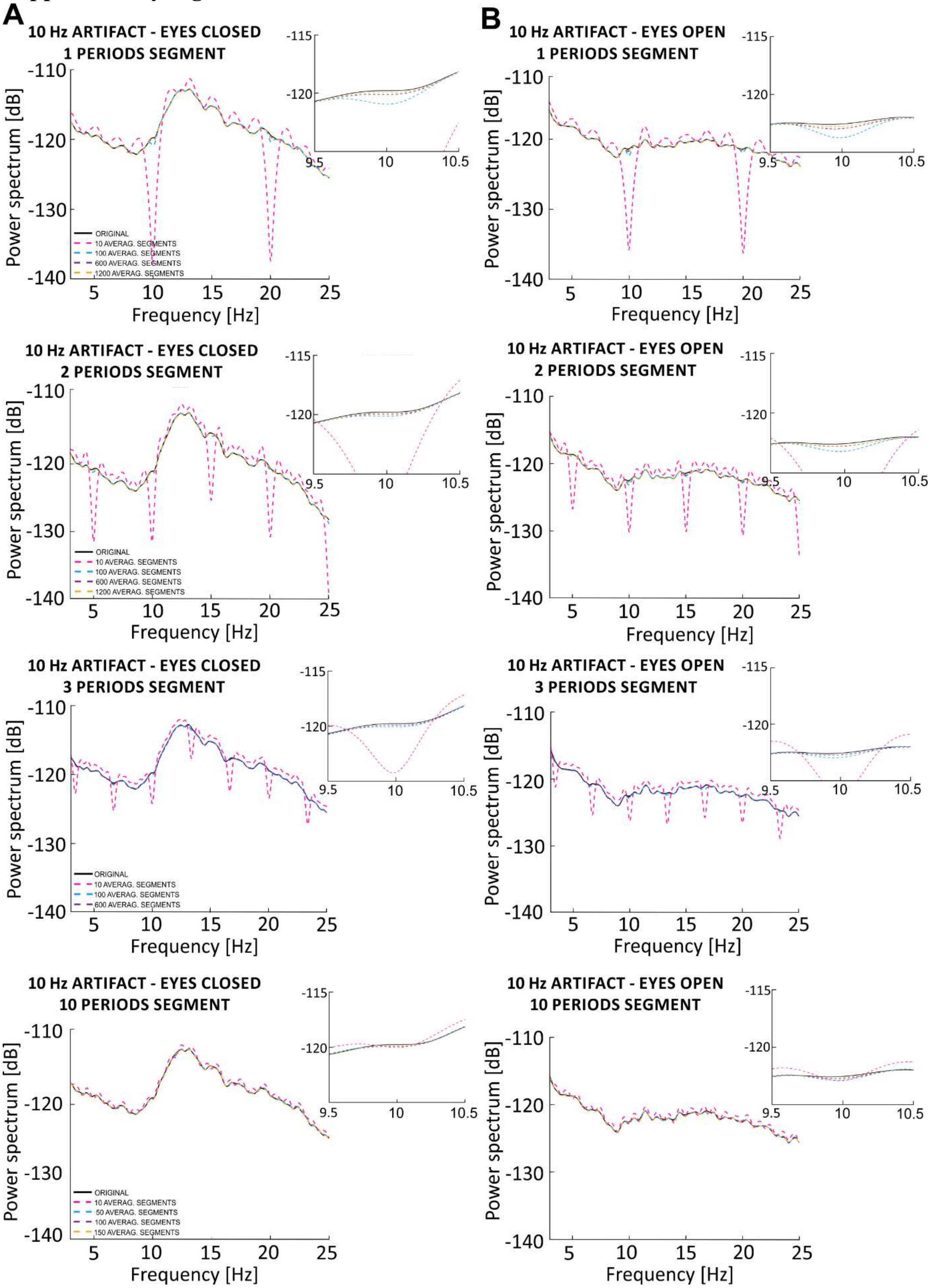

Supplementary Figure 7

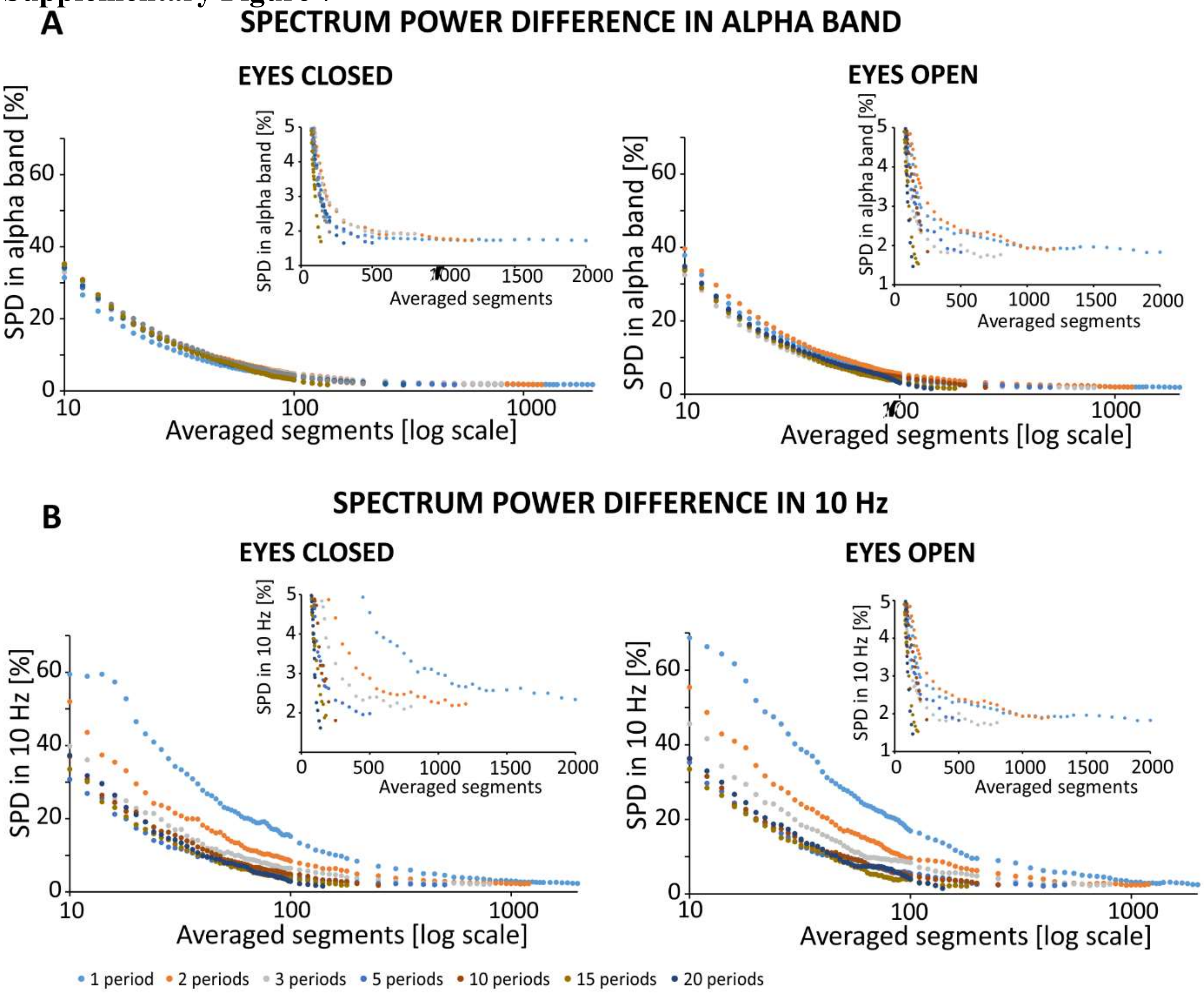

Supplementary Figure 8

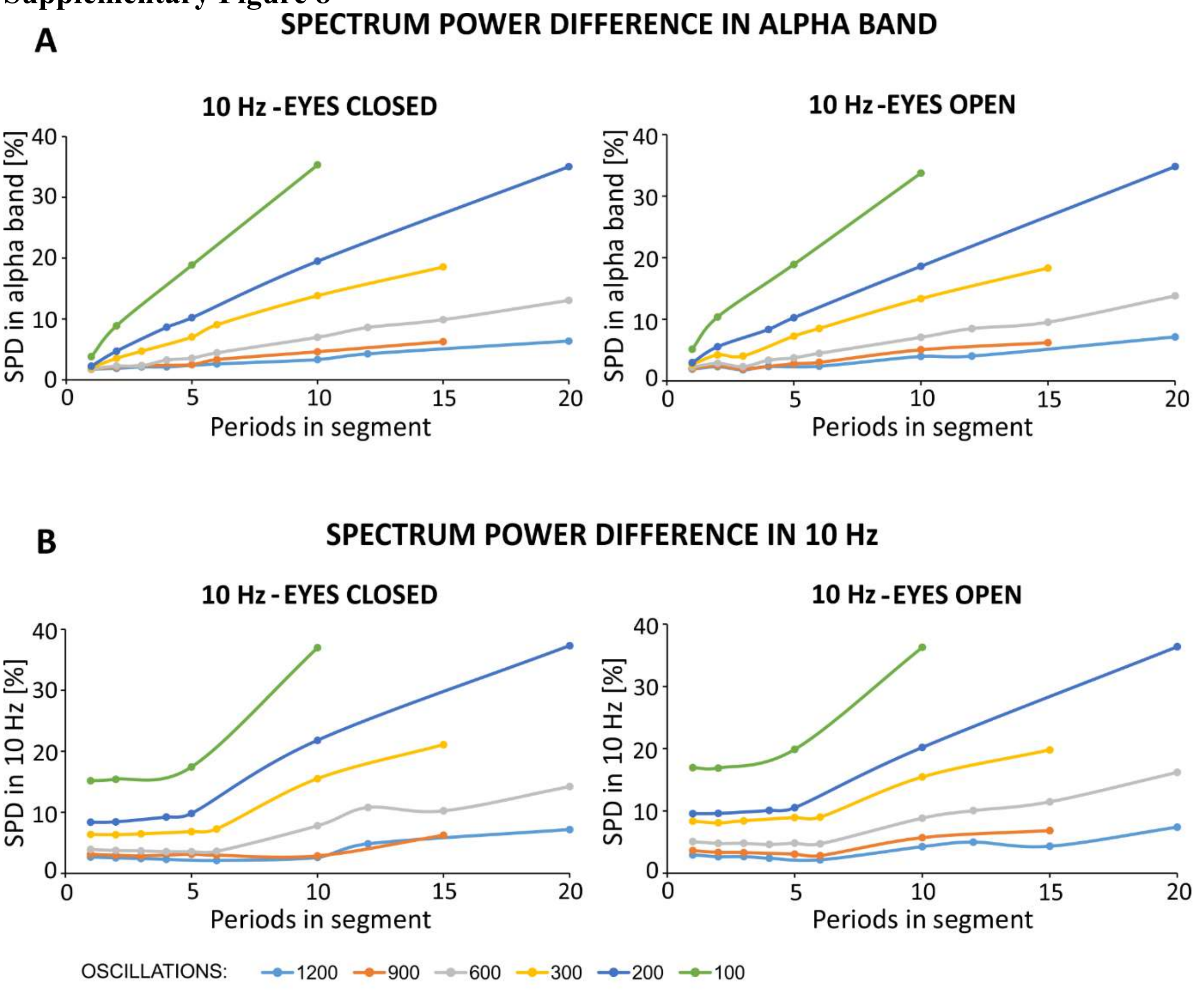

Supplementary Figure 9

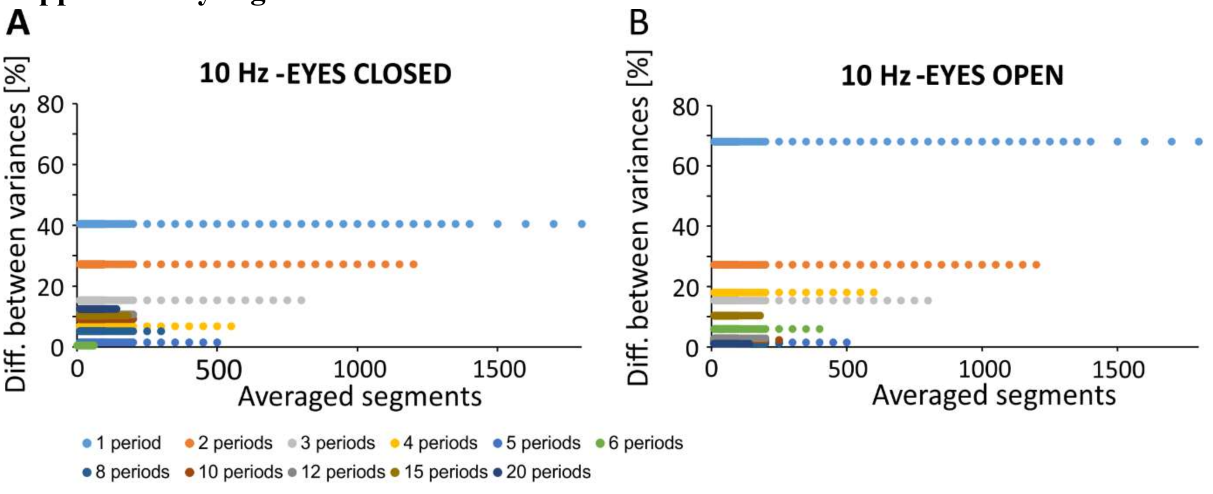

Supplementary Figure 10

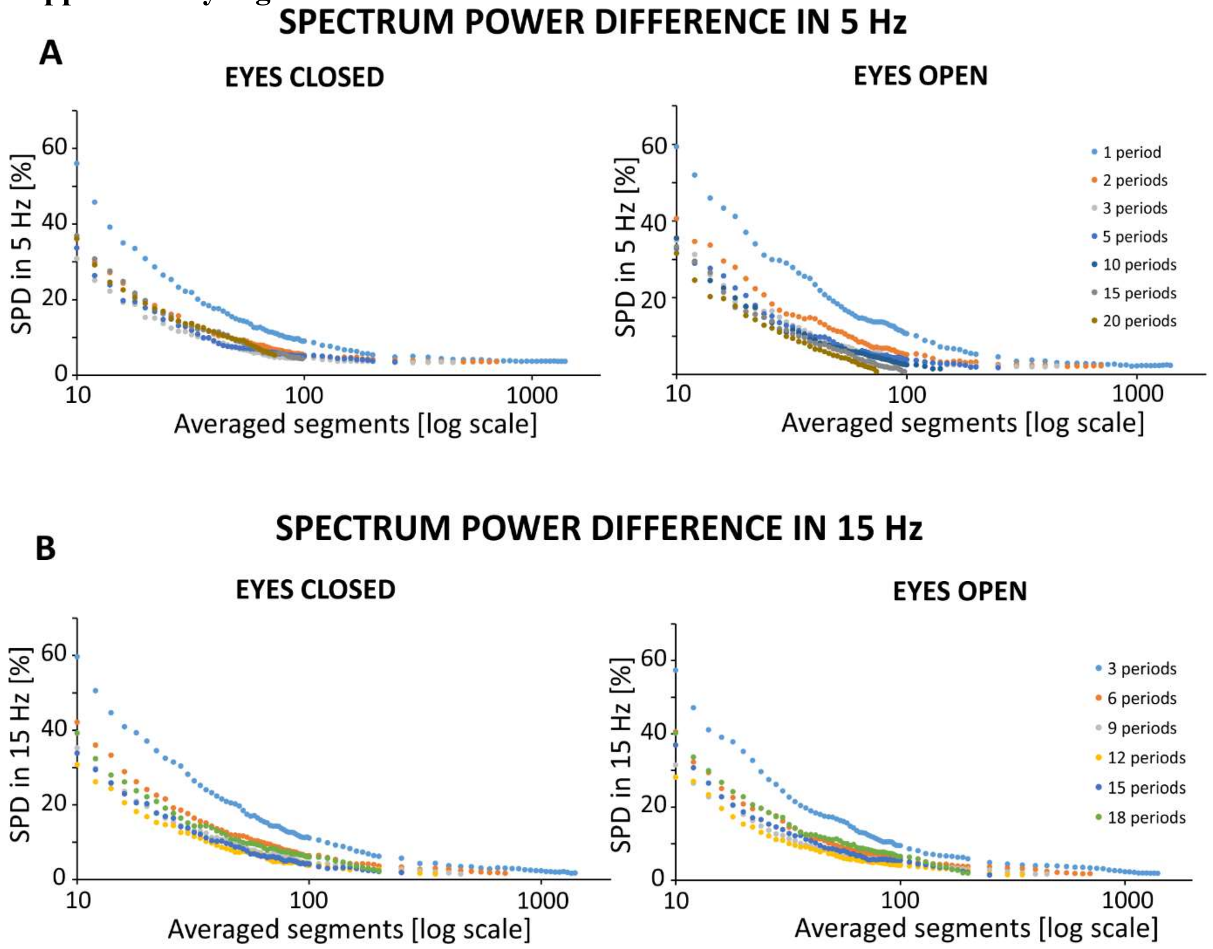

Supplement: Supplementary file 1 [file Data_Sheet_1.pdf]
